# De novo assembly and characterization of the liver transcriptome of *Mugil incilis* (lisa) using next generation sequencing

**DOI:** 10.1038/s41598-020-70902-5

**Published:** 2020-08-18

**Authors:** Angela Bertel-Sevilla, Juan F. Alzate, Jesus Olivero-Verbel

**Affiliations:** 1 Environmental and Computational Chemistry Group, School of Pharmaceutical Sciences, Zaragocilla Campus, University of Cartagena, 130015 Cartagena, Colombia; 2 Centro Nacional de Secuenciación Genómica—CNSG, Sede de Investigación Universitaria-SIU, Universidad de Antioquia, Medellín, Colombia

**Keywords:** Next-generation sequencing, Sequence annotation

## Abstract

*Mugil incilis* (lisa) is an important commercial fish species in many countries, living along the coasts of the western Atlantic Ocean. It has been used as a model organism for environmental monitoring and ecotoxicological investigations. Nevertheless, available genomic and transcriptomic information for this organism is extremely deficient. The aim of this study was to characterize *M. incilis* hepatic transcriptome using Illumina paired-end sequencing. A total of 32,082,124 RNA-Seq read pairs were generated utilizing the HiSeq platform and subsequently cleaned and assembled into 93,912 contigs (N50 = 2,019 bp). The analysis of species distribution revealed that *M. incilis* contigs had the highest number of hits to *Stegastes partitus* (13.4%). Using a sequence similarity search against the public databases GO and KEGG, a total of 7,301 and 16,967 contigs were annotated, respectively. KEGG database showed genes related to environmental information, metabolism and organismal system pathways were highly annotated. Complete or partial coding DNA sequences for several candidate genes associated with stress responses/detoxification of xenobiotics, as well as housekeeping genes, were employed to design primers that were successfully tested and validated by RT-qPCR. This study presents the first transcriptome resources for *Mugil incilis* and provides basic information for the development of genomic tools, such as the identification of RNA markers, useful to analyze environmental impacts on this fish Caribbean species.

## Introduction

Industrialization and urbanization are the main causes of aquatic ecosystem degradation, discharging a wide variety of pollutants that promote deleterious effects on organisms, with typical responses such as inhibition of growth, alterations in sexual maturation (reproductive capacity) and immunodeficiency^1–3^. The problem is magnified when aquatic organisms like fish, accumulate pollutants directly from water or sediment, and indirectly through the food chain^4^. Therefore, fish can serve as useful indicators of aquatic pollution as they play multiple roles across the trophic web, bioaccumulate chemical substances, and respond to low concentrations of toxicants^5,6^.

Mugilidae (mullets) is a fish family widely distributed in tropical and subtropical waters around the globe, particularly in coastal and estuarine areas where they play an important ecological role, and provide biomass to support fisheries^7–9^. Over the past years, Mullets have been proposed as pollution bioindicators for environmental degradation^10^. Mugilids, in particular species of genus *Mugil*, such as *Mugil cephalus* and *Mugil incilis*, have been extensively employed on environmental monitoring programs, as well as in toxicological studies in coastal zones impacted by human activities^1,10–16^. The use of these mugilids as sentinel species in these coastal systems arises from their wide geographical distribution; great abundance, salinity tolerance and bioaccumulation of land-based pollution, a feature largely enhanced by their consumption of benthic sediments along with their food^1,17^.

*Mugil incilis*, also known as mullet, lisa and lisa rayada, is one of the most abundant fish in the Caribbean. It is found in the western Atlantic Ocean, from Panama and Haiti to southeastern Brazil^18^. It can be found both in brackish estuaries and in marine and hyper-saline waters^19^. Juvenile fish (< 25 mm) are primarily planktonic or carnivorous feeders, whereas larger specimens switch their diet to detritus and benthic microalgae, ingesting large amounts of organic matter, sand or mud from the sediments^20^. These fish are in high demand in commercial and recreational fishing due to their high protein and vitamin content, being an important source of food in many countries^15^. However, there are several studies that report this fish as an intermediate host of several parasites, which contrasts with its nutritional importance^21^. In Colombia, *M. incilis* is one of the most widely distributed fish in the Caribbean coast^13,22^.

Along the Caribbean coast, Cartagena Bay is considered one of the ecosystems with high economic and environmental interest, as it hosts a great diversity of biological resources, but in the other hand, it receives many anthropic pressures, especially from industrial activities, such as oil refinery, pesticide packaging, metallurgical industry, and boatyards, including also naval and commercial shipping harbors. The chemical contamination of the bay has been the result of continuous, direct and indirect discharges of urban and industrial waste^13,23,24^. Some studies with native species of this ecosystem, such as *M. incilis* (lisa), have shown anthropic contamination by chemical substances, both organic and inorganic, and biological stressors, such as parasites. Heavy metals, such as mercury, have been detected in this species^13,25^, as well as polycyclic aromatic hydrocarbons (HAPs) and perfluorinated octyl sulphonates (PFOS) in bile^14,23^, and organochlorine pesticides in muscle (β-HCH, Aldrin, 4, 4′-DDE and endosulfan^16^. This species has also been found parasitized with nematodes of the Anisakidae family, in particular *Contracaecum* sp., as well as with Heterophydae trematodes, specifically *Ascocotyle (Phagicola) longa* in hepatic tissue^21,26,27^.

As presented before, most studies on *M. incilis* from the Cartagena Bay environmental have included measurements of pollutant levels in different tissues. However, a better understanding of the impact of this pollution requires additional approaches to correlate the chemical or biological exposure with the molecular and physiological responses in these organisms, necessary to generate reliable information on their current health status and the effects of exposure to different pollutants^6,28^. One of those approaches is the analysis of gene expression, a tool that provides multiple possibilities to evaluate molecular changes in exposed organisms^29,30^. The understanding/comprehension of the biological response related to chronic exposition to environmental pollutants at the transcriptomic level is essential to safeguard the adaptive potential of populations under heavy anthropogenic pressure^31–33^. For example, de novo assembly of transcriptomes from important aquaculture species, such as *Epinephelus coioides*, *Larimichthys crocea*, *Scophthalmus maximus*, *Lateolabrax japonicas* and *Labeo rohita* (Hamilton) has revealed a huge number of molecular markers relevant in the immune response after exposition to pathogen microorganisms^34,35^. Although experimentally controlled populations are ideal for evaluating the transcriptional profile during exposure to specific environmental stressors^36^, the study of toxic effects and transcriptional response in natural populations is essential for understanding the synergistic effects of multiple environmental stressors under field conditions^29,30^.

In fish, liver has been the focus of several toxicological studies and represents an essential organ for biomonitoring purposes^1,12,37^, mainly because it is sensitive to pollutant exposure, and it allows the assessment of hepatic damage and responses. Furthermore, its role in detoxification, nutrient synthesis and the transformation and storage of metabolites, represents complex biological functions that are essential for the survival of the organism^38,39^. However, the analysis of fish liver transcriptomes has been limited to only a few species and to our knowledge; no studies have been reported on the liver transcriptome of *M. incilis* or in other mugilid species. The purpose of this study was to characterize the hepatic transcriptome of *M. incilis* in order to facilitate future studies on gene expression related to the effects of environmental pollution in this Caribbean wild species.

## Results

### De novo assembly of the *Mugil incilis* hepatic transcriptome

Approximately 32,082,124 raw read pairs from the HiSeq platform were generated from the liver transcriptome sample from *M. incilis*. After cleaning with a Q30 base quality threshold, 31,061,840 read pairs remained. The clean read dataset was further assembled using Trinity software into 93,912 contigs larger than 200 bases that reached 92 Mb. Contig length ranged between 201 and 17,447 bases with an N50 value of 2,019 bases (Table 1). In order to identify contigs that belong solely to fish species, BLASTn and BLASTx comparison of the complete contig dataset was carried out and, using the software MEGAN, contigs were filtered according to its classification and only those belonging to Chordata were kept for further analysis. This filtered contig dataset that includes 40,296 contigs was considered from *Mugil incilis* (Table S1, Fig. S1).

**Table 1:** Summary of the assembled transcriptome of *M. incilis.*

Item	Number
Total length of sequence	91,495,553 bp
Total number of sequences	93,912
Average contig length	974 bp
Largest contig	17,447 bp
Shortest contig	201 bp
N25 stats	25% of total sequence length is contained in the 4,329 sequences > = 3,634 bp
N50 stats	50% of total sequence length is contained in the 12,855 sequences > = 2,019 bp
N75 stats	75% of total sequence length is contained in the 30,528 sequences > = 786 bp
Total GC count	43,537,515 bp
GC %	47.58%
Number of Ns	0
Ns %	0.00%

Ns, ambiguous bases.

The completeness of the *M. incilis* transcriptome assembly was quantified by comparing against a dataset of 3,640 actinopterygii single-copy orthologs (actinopterygii_odb10) using the BUSCO gene mapping method (BUSCO, RRID:SCR 015,008) for transcriptome assembly validation. This assessment reveals 61.0% completeness with the BUSCO Vertebrata gene set (48.9% complete single-copy BUSCOs, and 12.1% complete duplicated BUSCOs), 8.8% fragmented BUSCOs, and 30.2% of the 3,640 single-copy orthologs were classified as missing from our assembly (Table S2).

### Similarity analysis of the *M. incilis* liver transcriptome

Species distribution analysis (Fig. S2) revealed that maximum percentage of *Mugil incilis* transcript contigs showed significant similarity with *Stegastes partitus* (13.4%), followed by *Amphiprion ocellaris* (7.9%), *Larimichthys crocea* (5.1%) and *Acanthochromis polyacanthus* (4.5%). In addition, 237 (0.6%) of the transcripts were annotated to other eukaryotes, i.e., mammals, teleost fishes, and few prokaryotes vertebrates. Notably, due to the scarcity of genome information, only 39 contigs (0.1%) were matched with the genes from mugilid species such as *Mugil curema*.

### GO annotation of the lisa liver transcriptome

**Figure 1:**
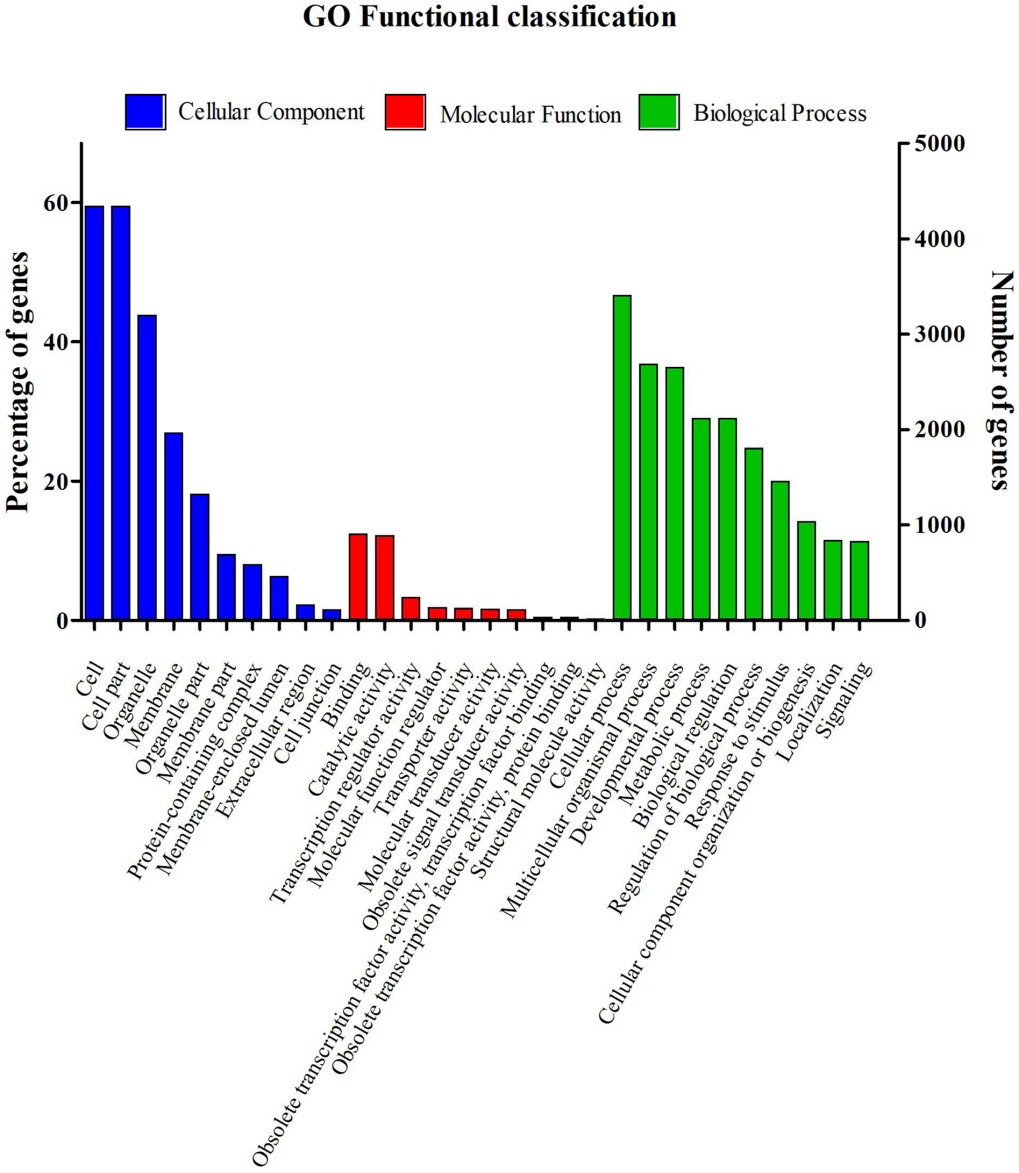
Function classifications of Gene Ontology (GO) terms of *M. incilis* transcriptome data. GO terms were annotated at level 2 of classification according to three main categories (Biological process, Cellular component and Molecular function). The *x*-axis indicates the subcategories, and the *y*-axis registers the percentages of genes (number of a particular gene divided by total gene number).

GO terms were assigned to assemble contigs and provided defined ontologies to express gene product properties. Among the 40,296 contigs detected in the liver transcriptome, a total of 7,301 sequences had GO term assigned, which were subsequently categorized into 93 functional groups (Fig. S3). GO analysis assigned 4,695 (40%) contigs to biological process, 5,069 (44%) to cellular component, and 1869 (16%) to molecular function. Briefly, for biological processes, genes involved in cellular process (GO:0009987) and multicellular organismal process (GO:0032501) were the most dominant categories; for molecular functions, binding (GO:0005488) were the most represented GO term, followed by catalytic activity (GO:0003824); while the principal categories observed in cellular component were cells (GO:0005623) and organelles (GO:0043226). We also identified genes involved in other major functions, such as developmental and metabolic processes, biological regulation, response to stimulus, and localization. The top 10 groups in the three main categories are shown in Fig. 1.

### Functional classification by KEGG

**Figure 2:**
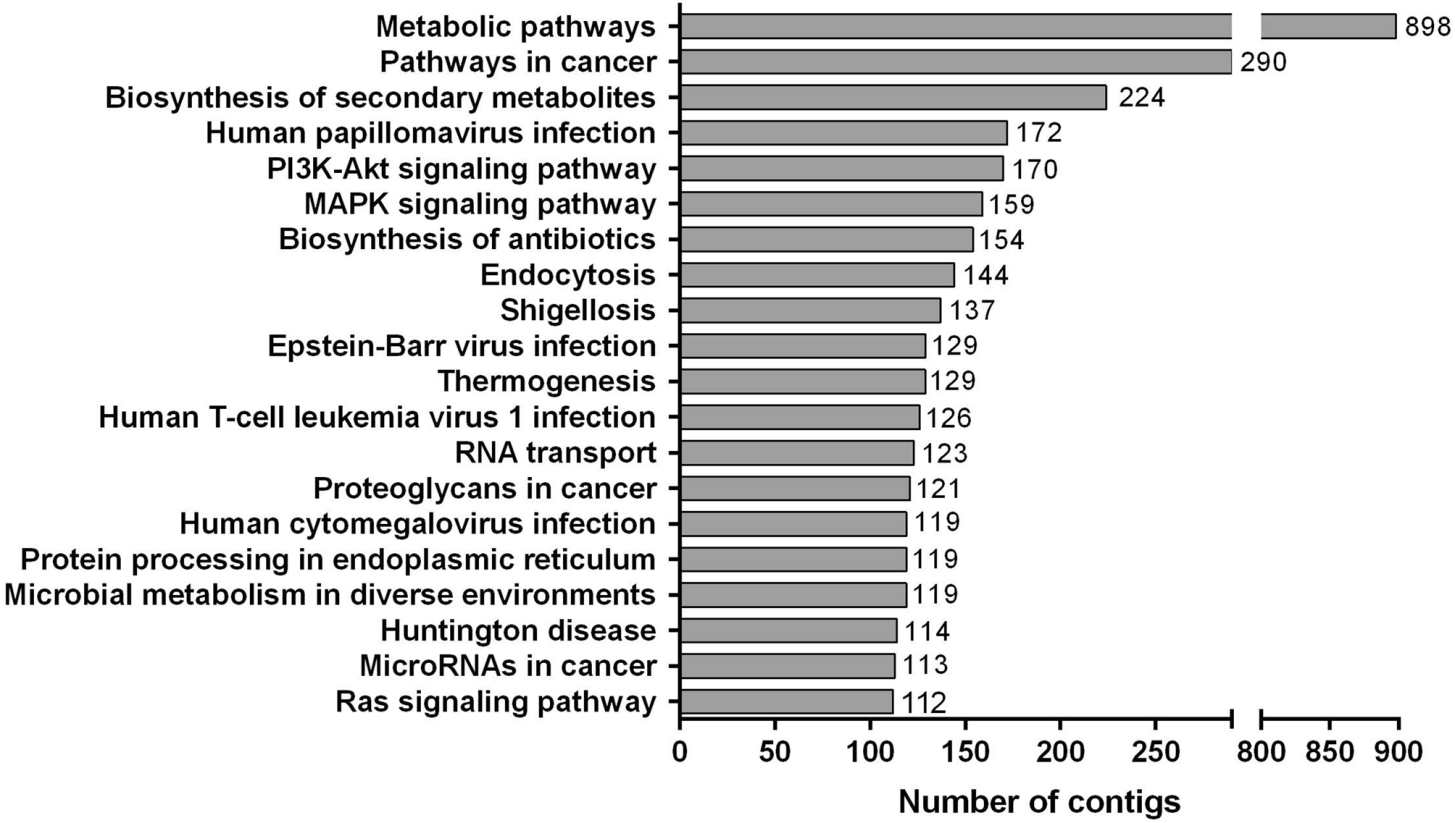
Top 20 pathways with the largest numbers of contigs based on KEGG annotation analysis.

To identify biological pathways in the liver of *M. incilis*, the contigs were mapped to the reference pathways recorded in the KEGG database. A total of 16,967 contigs were annotated in KEGG and located to 401 known KEGG pathways (Table S3). The top 20 pathways with the largest numbers of contigs are shown in Fig. 2. Among the 7 predicted KEGG pathways, the metabolic pathways represented the largest group, consisting of 898 contigs (5.29%), followed by cancer (290, 1.71%), biosynthesis of secondary metabolites (224, 1.32%), human papillomavirus infection (172, 1.01%) and PI3K-Akt signaling (170, 1.00%) (Fig. 2 and Table S3). These 401 pathways were subjected to six categories: human diseases (5,238, 30.9%), organismal systems (3,342, 19.7%), metabolism (3,269, 19.3%), environmental information processing (2,365, 13.9%), cellular processes (1,665, 9.8%), and genetic information processing (1,088, 6.4%) (Table S4).

### Validation of primers for real-time qPCR assays

The de novo sequencing of the hepatic transcriptome of *M. incilis* generated sequence knowledge for a species with virtually no available genomic information. PCR primers of a set of known target genes related to responses to xenobiotic exposure were generated from the transcriptome sequences. Twenty primers pairs were analyzed, including gene markers of heavy metal exposure, xenobiotic metabolism, nuclear receptor modulation, oxidative stress, DNA damage, inflammation, and lipid metabolism. Accession numbers and primer sequences for the target genes are listed in Table 2.

**Table 2:** Primer sequences for real-time PCR.

Gene name	Gene abbreviation	Gene ID	Forward (5′ to 3′)	Reverse (5′ to 3′)	Amplicon size (pb)
**Oxidative stress**					
Superoxide dismutase 1	SOD1	MH716024	TGGAGATAACACGAACGGGTG	ATTGGGGCCAGCGTGATTC	76
Catalase	CAT	MH716025	AAAGAGTGGTGCATGCCAAG	TGCCGACATGTTCAAACACC	102
Proteína de shock térmico de 70 KDa	HSP70	MH716026	CTGGATGTGACCCCCTTGTC	GTTGGGATGGTGGTGTTCCT	86
Factor Inducible por Hipoxia-1, Subunidad Alfa	HIF1A	MH716027	TAGTTGAGAGCAGCCAACCC	CAGCTCTGCACACTTGTCCT	80
**Exposure to xenobiotics**					
Cytochrome P4501A	CYP1A1	MH716028	TGTTCTTGGCCATCCTCGTC	CAGTGGTCATCCCTCACTCG	142
Cytochrome P450, family 3, subfamily A	CYP3A	MK110662	GACTCTCCTGGTTGCGTTCA	CCCGTACTTCTTGTGGCACT	172
Retinoid X receptor alpha isoformX2	RXRA X2	MK110663	TTCAACCCAGACTCCAAAGG	CGGGATACTTGTGTTTGCAG	103
Aryl hydrocarbon receptor	AHR	MH716029	CTCGGCTTTCACCAGACTGA	CAAGATGAGGTGGACTCGGC	161
**Apoptosis/necrosis**					
Apoptosis Regulator	Bcl-X	MH716030	GAGTTTCAAGAAGTGGCTGCTG	ACAGTCGTTTCTGAGCGAAG	83
Apoptosis Regulator BAX	BAX	MH716031	AGGCGATCAAGGAAATGCCA	GTGCGAGCTTCTTGTGGTTG	167
Caspase-3	CASP3	MK183035	TCAGAGAAATGGCACAGACG	TCATTTGCTCGACTGACTGG	104
Growth arrest and DNA damage inducible alpha	Gadd45A	MK183036	TTCACTTCACCCTCATCCAG	TTCACCCCCACTCTGTTTTC	125
Tumor necrosis factor alpha induced protein 3	TNF-α	MH716032	ATGCTAGAGGGCTACTGCGA	ATAACCAGAGGTGGGGCGA	80
**Immune activation/inflammation**					
Interleukin-6	IL6	MK183037	GAAAAACAAGAAGCGGGTCA	GCTTCTCCCTTCTGTCGATG	173
Major Histocompatibility Complex, Class II, alpha chain	MHC- class II alpha	MK183039	TTGGTCTGACTCTGGGTTTG	AGCTGCACTCGTTTCCTTTG	72
**Lipid metabolism**					
Peroxisome proliferator-activated receptor alpha	PPARA	MK183038	GAGGACTCGGTGTTGGACAG	CGTCGATGGACTGGGAGATG	91
Peroxisome proliferator-activated receptor gamma	PPARG	MH716033	CGGAGACAACATGCCTTTCG	CCTGAAACTCCGTGTGCAGT	138
**Housekeeping**					
Ribosomal Protein L13a (RP-L13)	L13a	MH712490	TCCTGCGTAAGAGGATGAACAC	TGGTTTTATGGGGCAGCATG	108
Elongation Factor-1 alpha	EF1	MH712491	CGTGAAGCTGATCCCACAGA	CCAGAGACCTCCTTGTACTCG	141
Glyceraldehyde 3-phosphate dehydrogenase	GAPDH	MH716023	AAGTCGGTATCAATGGATTTGGC	TCCACCTTCTTGGAGACGAA	73

**Figure 3:**
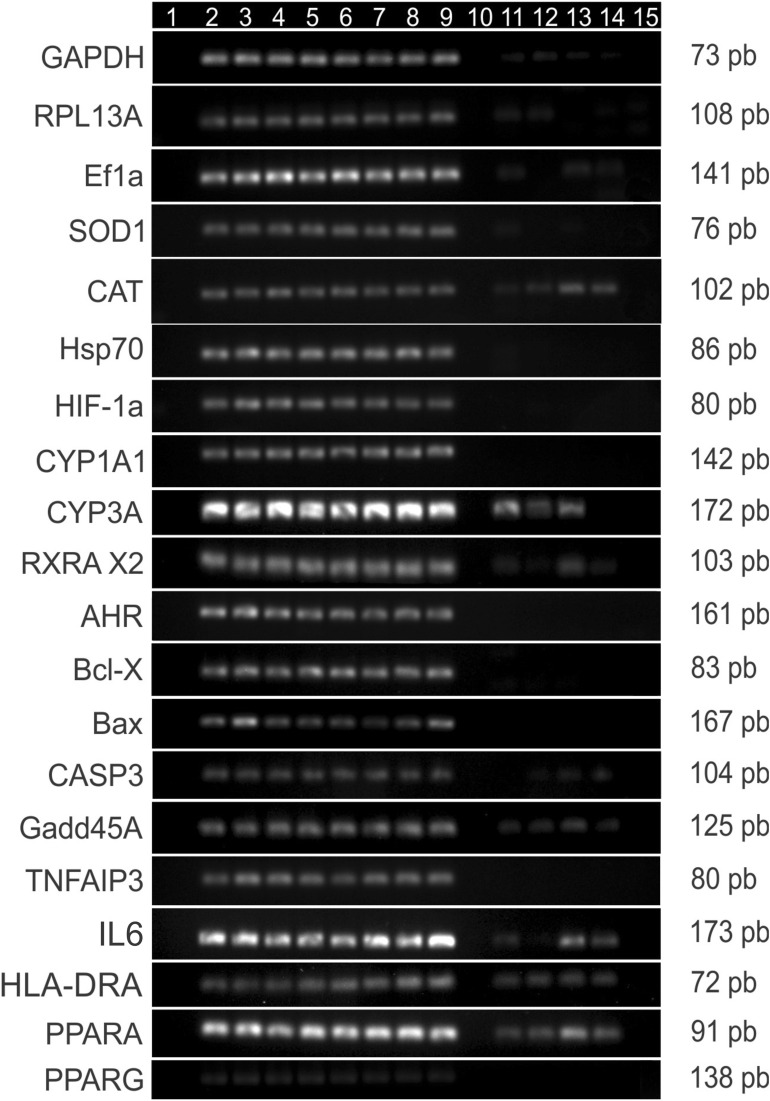
Gel electrophoresis of the RT-PCR products of genes associated with stress responses/xenobiotic detoxification. The lanes correspond to blank (1), cDNA from *M. incilis* (2–9), *P. magdalenae* (11–12), *H. malabaricus* (13–14) and *M. musculus* (15). All the gels were run under the same experimental conditions and are presented using cropped images. The entire gel photos of all genes evaluated can be found in Figure S5.

The C_T_ (Threshold cycle) is the cycle number at which the fluorescence generated within a reaction crosses the threshold line. When C_T_ values were used to generate a log-linear regression plot, the standard curve for the target genes showed a strong relationship (R^2^ ranging from 0.95 to 1; PCR efficiency ranging from 89 to 116%). A correlation coefficient greater than 0.99 shows good primer efficiency, and indicates a successful real-time PCR experiment. It is considered that a slope of the regression curve between − 3.9 and − 2.9 corresponds to PCR efficiencies ranging from 80 to 120%^40,41^. For all the genes evaluated in this work we found efficiency percentages ranging from 90 to 110%. A melting curve analysis showed no indication of primer-dimerization for the tested candidate oligonucleotides. PCR efficiencies for each primer pair are shown in Table 3. Furthermore, the primers specificity for target genes was confirmed by gel electrophoresis of the PCR products. All the primer sets amplified one single band of the expected fragment, and most of them showed specificity for targets from *M. incilis*. Some of the primers, including housekeeping genes, amplified fragments from related fish species such as *P. magdalenae* and *H. malabaricus*. The electrophoresis of PCR products for the evaluated genes is presented in Fig. 3.

**Table 3:** PCR primer efficiencies used in Real-time qPCR assays*.*

Genes and pathways	Amplification efficiency (%)	R^2^
**Oxidative stress**		
SOD1	107.8	0.99
CAT	93.5	1.00
HSP70	112.8	0.98
HIF1A	100.3	1.00
**Exposure to xenobiotics**		
CYP1A1	99.7	1.00
CYP3A	90.1	1.00
RXRA X2	93.7	1.00
AHR	116.6	0.99
**Apoptosis/necrosis**		
Bcl-X	100.9	1.00
BAX	116.1	1.00
CASP3	101.8	1.00
Gadd45A	106.0	0.95
TNF-α	113.3	0.98
**Immune activation/inflammation**		
IL6	89.6	0.99
HLA-DRA	91.8	0.99
**Lipid metabolism**		
PPARA	94.2	1.00
PPARG	91.2	1.00
**Housekeeping**		
L13a	93.0	1.00
EF1	96.0	1.00
GAPDH	91.7	1.00

## Discussion

The accelerated development of high-throughput sequencing technology has revolutionized our way of studying genetic diversity and gene expression. In this road, the RNA-Seq technology has become the method of choice for the study and characterization of dynamic transcriptomes, and the de novo transcriptome assembly provides a powerful research tool for differential transcriptome analysis in fish species^42^. In the past several years, transcriptomic studies in teleost fish have increased, leading to a tremendous pool of genetic knowledge (e.g. see database FISHIT [https://www.fish-it.org/hcmr] for 20 transcriptomes). However, although Colombia is considered one of the most biodiverse countries in the world, there is little knowledge on fish species commonly found in its tropical ecosystems. The assembly of the transcriptome of *Mugil incilis* opens a large window of opportunities to develop toxicological studies with this organism, which can be found along the Caribbean.

Some reports on transcriptome data for Mugilids have been focused on population genetic structure^43^ and innate defense of the host against pathogens^44,45^. In this study, the transcriptome of hepatic tissue from *M. incilis* was generated using Illumina HiSeq 2000 sequencing platform. A total of 93,912 contigs were assembled with an average length of 974 bp, a greater value than those obtained in previous studies using other de novo assembly methods for teleosts, such as viviparous blenny (*Zoarces viviparus*; 395 pb)^46^, Guppy (*Poecilia reticulata*; 464 pb)^47^, European eel (*Anguilla;* 531 pb)^48^ and common carp (*Cyprinus carpio*; 888 pb)^49^. All this transcriptome sequencing data may be sufficient and necessary to discover new genes and create new tools for the analysis of gene function and expression in *M. incilis*.

To evaluate the quality and completeness of our transcriptome assembly we used the N50 reference-free metric and the reference-based BUSCO metric, which give us an idea of how good our assembly is in terms of continuity and coverage of the contigs respectively. The N50 measure showed that 50% of total sequence length is contained in sequences equal or greater than 2,019 bp, which is not a short length for the transcripts in our transcriptome assembly^47,49,50^. On the other hand, the BUSCO analysis showed that most of the Actinopterygian single-copy orthologs (61%) were found completely in our assembly and 30.2% of the 3,640 orthologs were classified as missing from our assembly. This seems to be a relatively good level of completeness, when comparing with other assemblies reported for this class of organisms, especially if we consider that BUSCO recovery tends to be higher when multiple tissues and different stages are used to generate the assemblies, and our assembly was made from a single tissue of a single specimen^50,51^. Furthermore, only 8.8% of the orthologs were recovered partially, indicating a low level of incomplete transcripts. We also ran a BUSCO analysis using the Vertebrata and Eukaryota datasets (see Table S2 in supplementary material), which showed a greater percentage of complete BUSCOs recovery (68% and 92.1% respectively) which may reflect evolutionary innovations in the *Mugil* genus.

Homology search for the clustered sequences was carried out using BLASTX against the NCBI non-redundant (Nr) database. The distribution of top hit species suggested that most of the annotated sequences corresponded to known nucleotide sequences of a small group of fish species, *S. partitus*, *A. ocellaris* and *L. crocea* (Fig. S2). It was not surprising that *M. incilis* contigs shared the highest percentage with *S. partitus*, as both of them belong to the order Perciformes, although such finding may be due to the availability of the complete genome of *S. partitus,* providing sufficient gene sequences and annotations for comparison analyses. However, the transcriptomic similarities between *M. incilis* and *S. partitus* only reached 13.4%, suggesting these two fish species might be distantly related in subfamilies of Perciformes, Mugilidae and Pomacentridae for *M. incilis* and *S. partitus*, respectively.

The GO functional annotation indicated genes involved in cellular process and multicellular organismal were the most represented categories for biological processes. For cellular components, the major category represented was cell. For molecular function, binding was the most strongly represented GO term, followed by catalytic activity, results that can be useful for gene functional studies. Furthermore, 16,967 contigs were annotated to 6 categories in KEGG, including cellular processes, genetic information processing, environmental information processing, metabolism, human diseases and organismal systems. Signal transduction had the most number of genes (2,007) in environmental information. For metabolism, global and overview maps had the most number of genes (1,572), whereas for organismal systems, the immune system comprises the most number of genes (1,045) (Table S4). In the present study, PI3K-Akt, MAPK, Ras and Rap1 signaling pathways were found to be the most highly enriched. These pathways are ubiquitous and crosstalk in all eukaryotic cells. MAPK signaling, for example, regulates a wide variety of cellular processes, including proliferation, motility, differentiation, stress responses, and apoptosis^52^; as well as cellular processes related to responses against diverse environmental stressors, such as heat, cold, UV radiation, reactive oxygen species, desiccation and pathogen attack^53^. The transcription of genes related to these signaling pathways suggested *M. incilis* is a sensitive model for physiological studies.

The 1,045 genes in the immune system were further grouped into 21 pathways (Fig. S4). NOD–like receptor signaling pathway and chemokine signaling pathway were the top two pathways with the greatest number of expressed genes. Nucleotide-binding oligomerization domain (NOD)-like receptors (NLRs) are a specialized group of cytoplasmic pattern recognition receptors (PRRs) that play an important role in the detection of various pathogens and regulation of innate immune responses. In fish, three distinct subfamilies of NLRs have been identified and characterized: NLR-A, NLR-B, and fish-specific NLR-C^54^. Expression of NLR-C subfamily (NLRCs) has been reported in many different teleost fish species such as zebrafish^55^, Japanese flounder^56^ and turbot^57^. These receptors are activated by a variety of bacterial pathogens or microbial ligands, including lipopolysaccharides (LPS), peptidoglycans (PGN) and polyinosinic-polycytidylic acid (Poly (I:C)), suggesting their participation in the fish innate immune response^54^. The other signaling pathway with greater annotation was related to chemokines, a superfamily of small size chemoattractant peptides that provide directional cues for cell migration and positioning, fundamental for protective host response. The chemokine-mediated immune response to pathogens involves not only leukocyte migration, but also promotes differentiation of recruited cells to trigger the first steps of the innate and acquired immune response^58^. Other important pathways include Fc gamma R-mediated phagocytosis, natural killer cell mediated cytotoxicity, leukocyte transendothelial migration, and T cell receptors, also critical in immune response. As *M. incilis* has been found parasitized with *Contracaecum* sp. and *Ascocotyle *(*Phagicola*)* longa* in the Colombian Caribbean^21,26,27^, the characterization of these pathways will be an essential tool to study the interaction fish-parasites in this species, especially considering the high selectivity obtained for designed primers (Fig. 3).

The transcriptome assembly of *M. incilis* generated in this work, as well as the PCR validation for several signaling pathways, will allow further molecular toxicology research on this species, as this fish has been used to monitor different chemical and biological pollutants in the Caribbean. The highly parasitized status of the fish, together with the different pollutant levels it may receive, creates a unique opportunity to study host-parasite interactions, and how environmental contaminants modulate this process.

## Materials and methods

### Sample collection and handling

One specimen of *M. incilis* (lisa) was obtained with the help of local fishermen in Cartagena Bay (10°24′ N 75°30′ W). The specimen was stored in a plastic bag and transported on ice to the laboratory for immediate processing. Prior to RNA extraction, the liver sample was frozen in liquid nitrogen and grounded into a fine powder using a pre-cooled mortar and pestle.

### RNA extraction, library construction and sequencing

RNA Extraction was performed according to the manufacturer's directions using Trizol reagent (Invitrogen, California, USA). RNA concentration and integrity of the sample was measured on an Agilent 2100 Bioanalyzer (Agilent Technologies) using an RNA Nano Bioanalysis chip. Mitochondrial RNA enrichment, library construction, and sequencing was conducted by Macrogen Inc. (Seoul, Korea) with Truseq mRNA kits and the Illumina HiSeq 2000 instrument, with 100 bp paired end (PE) reads.

### Sequence data processing and de novo assembly

Read quality was controlled using trimming threshold of Q30 bases with the program PRINSEQ^59^. After the quality filter, reads with less than 50 bases were rejected. The assembly step was carried out with the program Trinity (https://trinityrnaseq.sourceforge.net) employing default settings^60^. After the assembly, only the contigs longer than 200 bases were kept.

### Functional contigs annotation and classification

Sequencing data were aligned using BLASTx alignment (E-value cut-off of 10^−5^) with protein databases, including NCBI non-redundant (Nr) database and KEGG database. BLASTx comparisons and the software MEGAN were used to filter contigs that belong to *Mugil incilis*. Since *Mugil* sequences are scarce in the database, all sequences classified as Chordata were considered as of *Mugil* origin. Fish (Chordata) filtered contigs were loaded into the eggNOG database (https://eggnog.embl.de) in order to obtain general gene ontology (GO) annotations. The contigs/transcripts annotation was done assigning the GO terms according to three main categories: molecular function; cell component; and biological process^61^, and the gene ontology annotation results were plotted using the WEGO software at GO level 2 with default parameters (https://wego.genomics.org.cn). The assignment of KEGG pathways to the transcripts was done through the online KEGG Automatic Annotation Sever (KAAS; https://www.genome.jp/kegg/kaas) using the bi-directional best-hit method. Several contigs/transcripts of the stress responses/detoxification and immune system were selected and annotated using the BLASTx results and the KEGG annotation. CDS prediction was carried out manually using Artemis visualizer (Sanger)^62^.

### Design of primers and validation for real-time qPCR assays

Validation of gene expression quantification in hepatic tissue of eight *M. incilis* specimens was conducted using real time quantitative PCR (RT-qPCR) for twenty genes associated with stress responses/detoxification (GAPDH, L13a, EF1, SOD1, CAT, HSP70, HIF1A, CYP1A1, CYP3A, RXRA X2, AHR, Bcl-X, BAX, CASP3, Gadd45A, TNF-α, IL6, MHC-II class alpha, PPARA and PPARG). Primers for each target gene were designed with “primer-BLAST” tool from Genbank (https://www.ncbi.nlm.nih.gov/tools/primer-blast) using the transcript sequences obtained from the transcriptome sequencing. The specificity of primers PCR was checked at the end of each amplification using a DNA melt curve analysis and by the observation of single amplification products of the expected size on agarose gels. Amplification efficiency was determined using a five-point 1:10 dilution series starting with cDNA corresponding to 100 ng of input total RNA^63^. Samples of cDNA from other fish species (*Prochilodus magdalenae* and *Hoplias malabaricus*) different from the mullets were included, as well as a sample of cDNA from *Mus musculus*, in order to verify the specificity of the primers. The specific primers used for RT-qPCR are listed in Table 2.

The total RNA was isolated from liver tissue using RNeasy®Mini Kit (Qiagen, California, USA) as described by the manufacturer. The RNA was quantified by spectrophotometry (A260) using a NanoDrop 2000 Spectrophotometer (Thermo Scientific), and the A260:A280 ratio (1.9–2.0) was calculated to verify the RNA purity. Furthermore, the integrity of RNA was assessed by visual inspection of 28S and 18S ribosomal RNA after electrophoresis on an agarose gel. One microgram of total RNA was reverse transcribed, for each sample, using QuantiTect® Reverse Transcription Kit (QiagenInc, Valencia, CA, USA). The complementary DNA was used as template in a 20 μL PCR reaction containing 10 pmol each of forward and reverse gene-specific primers and 2 × SYBR® Green qPCR Master Mix (Thermo Scientific). Real time-PCR reactions were run in a StepOnePlus Real-Time PCR System (Applied Biosystems, Foster City, CA), under the following conditions: Initial denaturation and enzyme activation for 10 min at 95 °C, followed by 40 cycles of 95 °C for 15 s (denaturation) and 60 °C for 1 min (annealing/extension). A melt curve analysis was performed after the 40th cycle (10 s at 95 °C, then 65–95 °C in 0.5 °C increments every 5 s). A negative control (without DNA template) was run in duplicate on each plate to verify the absence of cDNA contamination^64^.

### Ethical statement

Protocols for the use of animals in this study were reviewed and approved by the Ethics Committee of the University of Cartagena (Act No. 123; 30-07-19). The field study did not involve endangered or protected species, and it was granted with permission to access genetic resources from the Ministry of Environment, Housing and Territorial Development from Colombia (Contract No. 148; 09-11-17). Conducted experiments were carried out in accordance with the International Guiding Principles for Biomedical Research Involving Animals (https://www.cioms.ch/frame1985textsofguidelines.html).

## Conclusion

This is the first report on the sequencing, de novo assembly, and functional annotation of the *Mugil incilis* transcriptome. The identification of the CDS sequences for several of these candidate genes will help developing primers for qPCR, which would be used to assess gene expression levels in mugilid species.

## Supplementary information

Supplementary information.

## Data Availability

The raw reads produced in this study were deposited in the NCBI database Sequence Read Archive (https://www.ncbi.nlm.nih.gov/Traces/sra) under the accession number SRP157095. This Transcriptome Shotgun Assembly project has been deposited at DDBJ/ENA/GenBank under the accession GHNU00000000.
