# Supplementary material for: De novo assembly and characterization of the liver transcriptome of Mugil incilis (lisa) using next generation sequencing
Source: Sci Rep. 2020 Aug 18;10:13957. doi: 10.1038/s41598-020-70902-5 (PMC7435268; doi:10.1038/s41598-020-70902-5)

Supplementary material

De novo assembly and characterization of the liver transcriptome of *Mugil incilis* (lisa) using next generation sequencing.

Authors

Angela Bertel-Sevilla[1], Juan F. Alzate[2], Jesus Olivero-Verbel[1]*.

[1]Environmental and Computational Chemistry Group, School of Pharmaceutical Sciences, Zaragocilla Campus, University of Cartagena, Cartagena, 130015, Colombia.
[2] Centro Nacional de Secuenciación Genómica—CNSG, Sede de Investigación Universitaria-SIU, Universidad de Antioquia, Colombia.

*. Corresponding author:

Jesus Olivero-Verbel.
Environmental and Computational Chemistry Group
School of Pharmaceutical Sciences
Zaragocilla Campus
University of Cartagena, Cartagena, 130015
Colombia.
Tel.: +57 (5) 6698179, 57 (5) 6698180; fax: +57 (5) 6698323.
E-mail: joliverov@unicartagena.edu.co

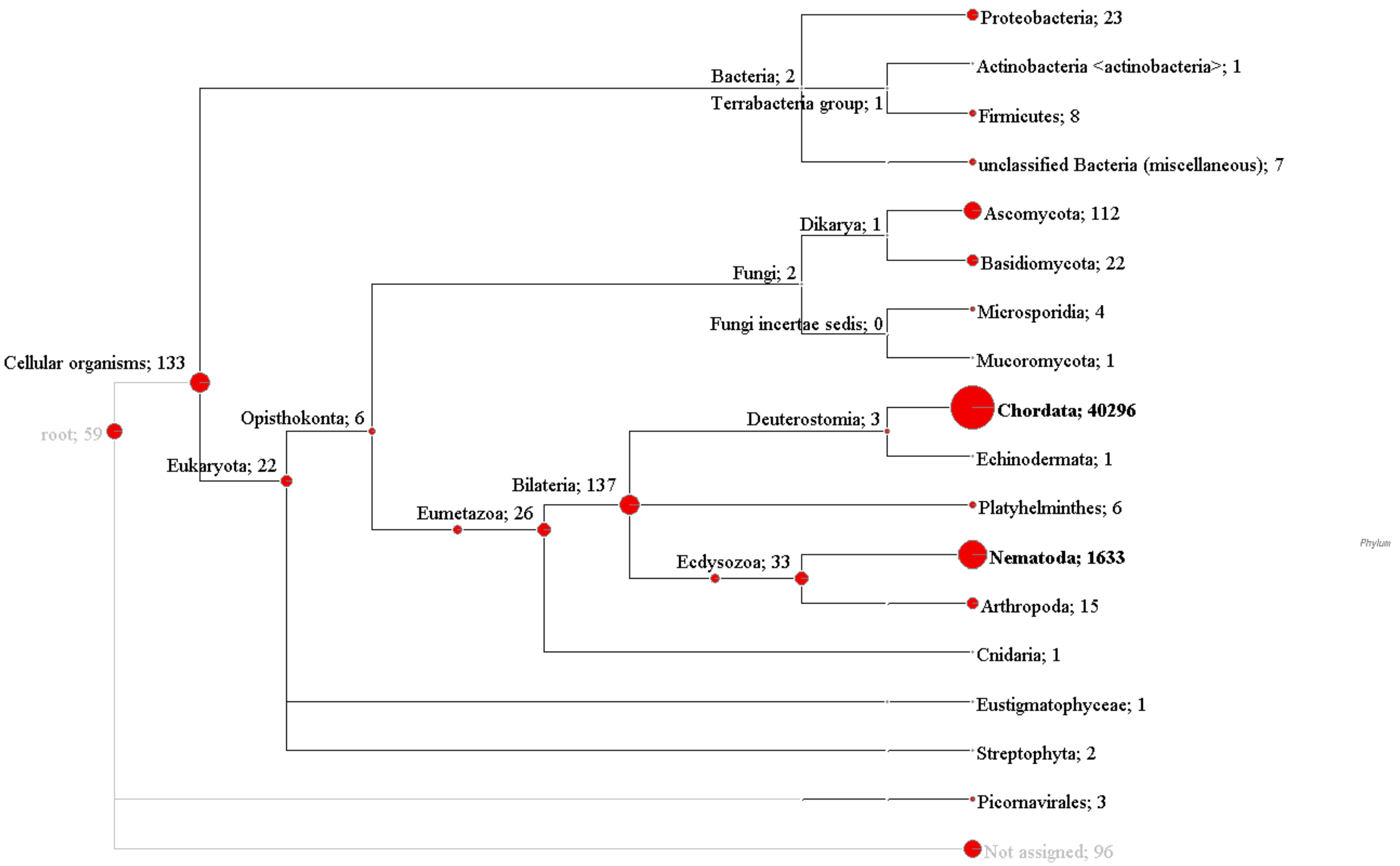

Supplementary Figure S1: MEGAN-LR taxonomic binning: nodes are scaled to indicate the number of reads contained in all assigned contigs in each bin.

Table S1. Summary of the statistics Chordata

Item	Number
Total length of sequence	67698952 bp
Total number of sequences	40296
Average contig length	1680 bp
Largest contig	17447 bp
Shortest contig	201 bp
N25 stats	25% of total sequence length is contained in the 2821 sequences >= 4.254 bp
N50 stats	50% of total sequence length is contained in the 7871 sequences >= 2.712 bp
N75 stats	75% of total sequence length is contained in the 15936 sequences >= 1.598 bp
Total GC count	33765.135 bp
GC %	49.88 %
Number of Ns	0
Ns %	0.00 %

Ns, ambiguous bases

Table S2. BUSCO statistics for the completeness of the *M. incilis* transcriptome assembly in comparison with the eukaryote, vertebrata and actinopterygii gene sets.

	**Orthologs datasets**		
	**Eukaryota_odb10**	**Vertebrata_odb10**	**Actinopterygii _odb10**
**Complete BUSCOs**	235 (92.1%)	2280 (68 %)	2221 (61%)
**Complete and single-copy BUSCOs**	188 (73.7%)	1827 (54.5%)	1781 (48.9%)
**Complete and duplicated BUSCOs**	47 (18.4%)	453 (13.5%)	440 (12.1%)
**Fragmented BUSCOs**	11 (4.3%)	380 (11.3%)	320 (8.8%)
**Missing BUSCOs**	9 (3.6%)	694 (20.7%)	1099 (30.2%)
**Total BUSCO groups searched**	255	3354	3640

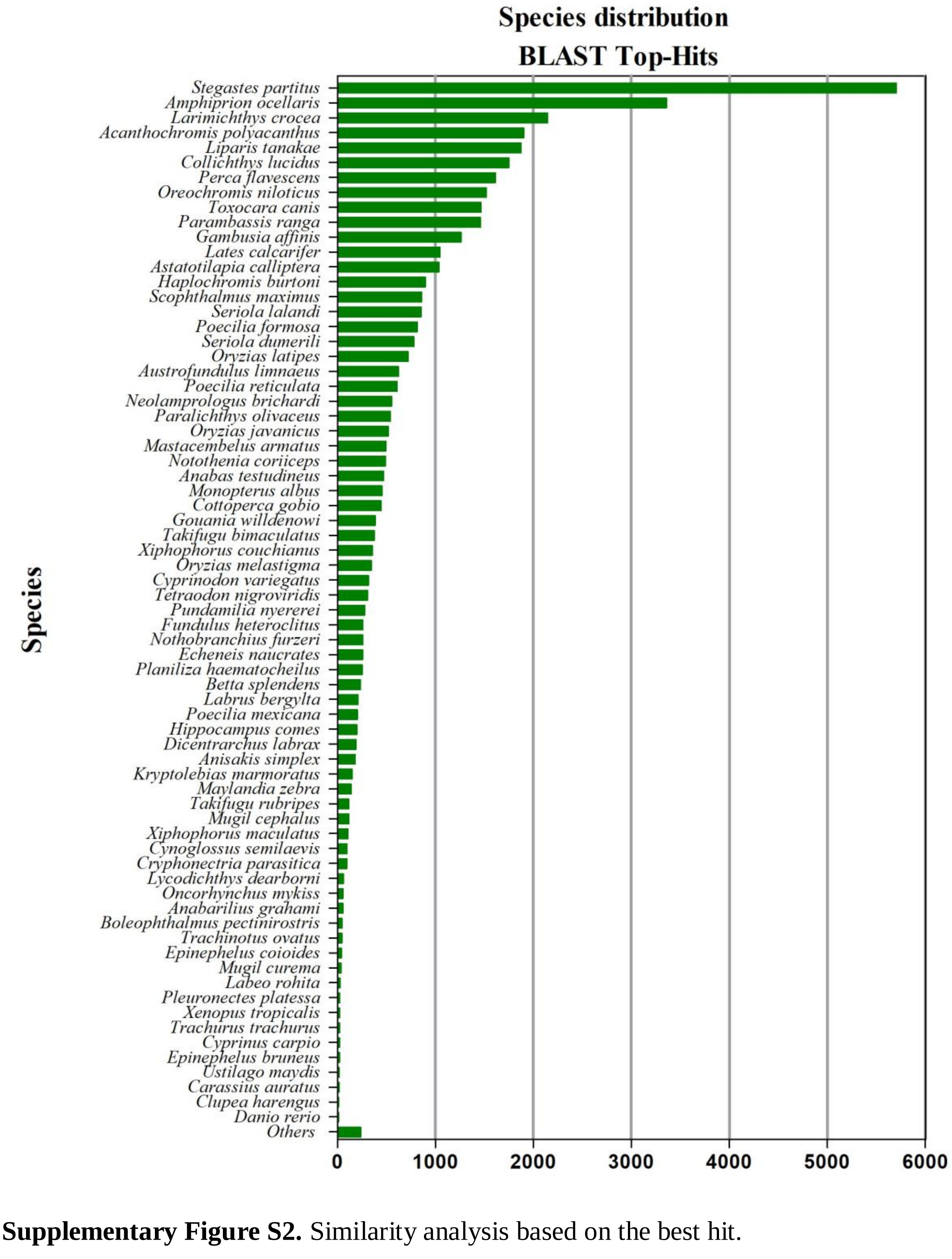

Supplementary Figure S2. Similarity analysis based on the best hit.

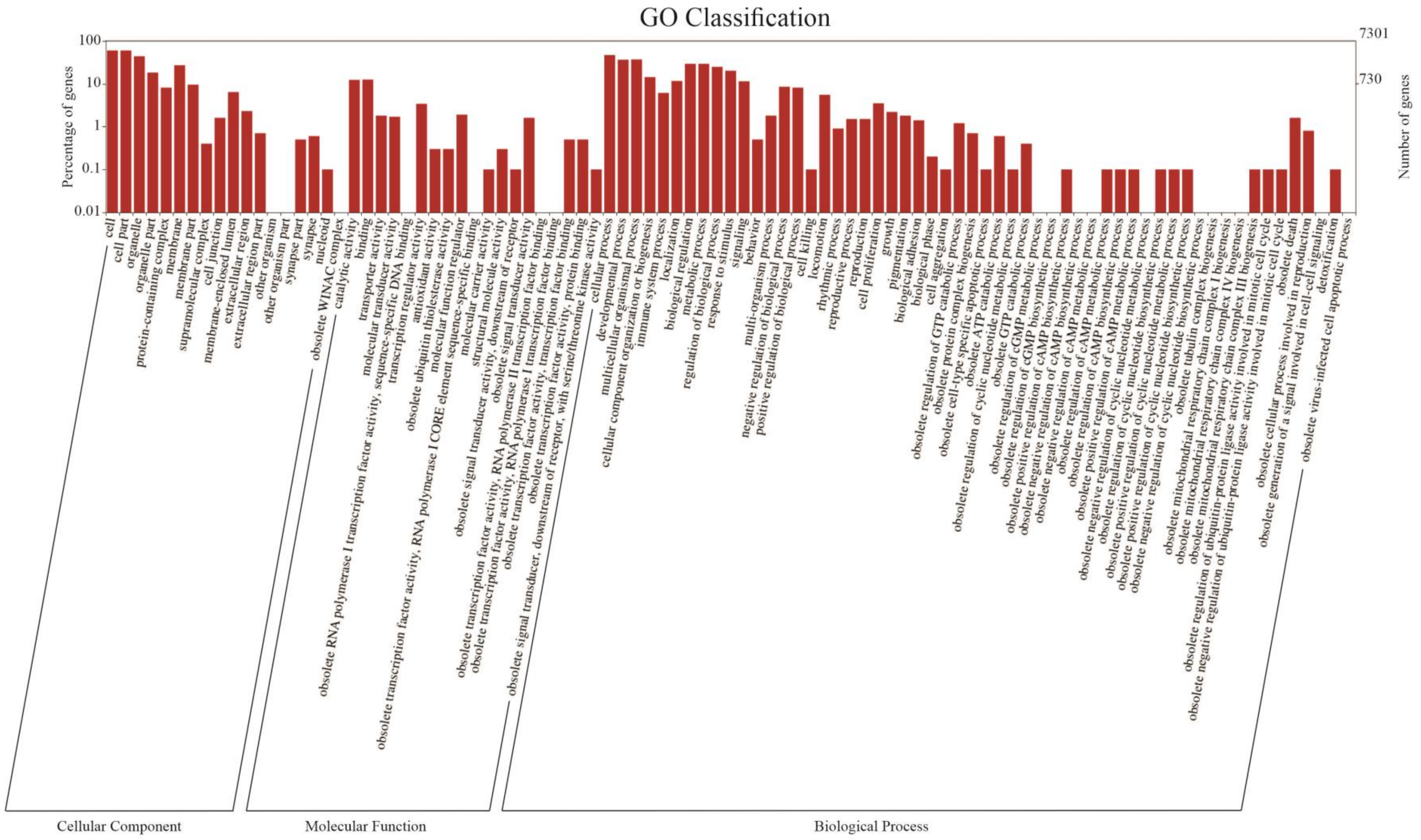

Supplementary Figure S3. Gene Ontology (GO) categories of the contigs. Distribution of the GO categories assigned to the *M. incilis* transcriptome. A total of 7.301 contigs were assigned to at least one GO term including biological process, cellular component and molecular function.

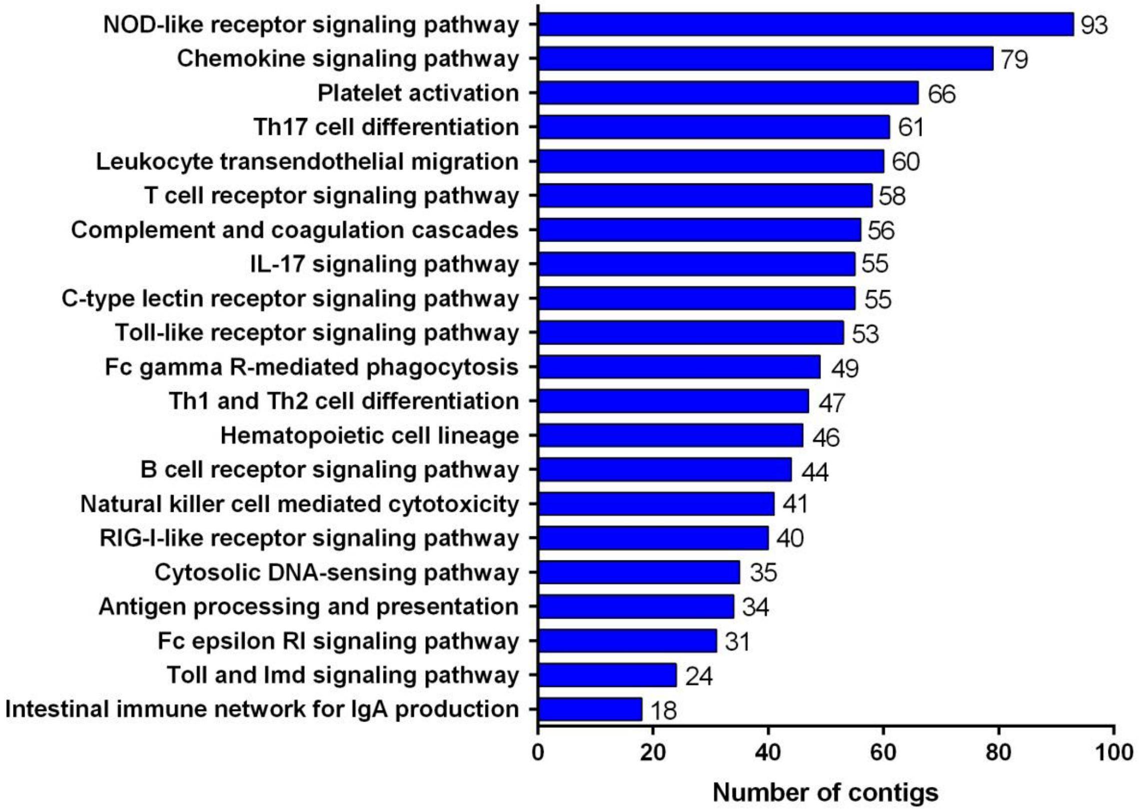

Supplementary Figure S4: Contigs related to immune pathways based on KEGG annotation analysis.

Supplementary Table S3. Pathway assignment based on KEGG.

#	Pathway	Number of contigs	Percent of contigs	Pathway ID
1	Metabolic pathways	898	5.29%	Ko 01100
2	Pathways in cancer	290	1.71%	Ko 05200
3	Biosynthesis of secondary metabolites	224	1.32%	Ko 01110
4	Human papillomavirus infection	172	1.01%	Ko 05165
5	PI3K-Akt signaling pathway	170	1.00%	Ko 04151
6	MAPK signaling pathway	159	0.94%	Ko 04010
7	Biosynthesis of antibiotics	154	0.91%	Ko 01130
8	Endocytosis	144	0.85%	Ko 04144
9	Shigellosis	137	0.81%	Ko 05131
10	Thermogenesis	129	0.76%	Ko 04714
11	Epstein-Barr virus infection	129	0.76%	Ko 05169
12	Human T-cell leukemia virus 1 infection	126	0.74%	Ko 05166
13	RNA transport	123	0.72%	Ko 03013
14	Proteoglycans in cancer	121	0.71%	Ko 05205
15	Microbial metabolism in diverse environments	119	0.70%	Ko 01120
16	Protein processing in endoplasmic reticulum	119	0.70%	Ko 04141
17	Human cytomegalovirus infection	119	0.70%	Ko 05163
18	Huntington disease	114	0.67%	Ko 05016
19	MicroRNAs in cancer	113	0.67%	Ko 05206
20	Ras signaling pathway	112	0.66%	Ko 04014
21	Cytokine-cytokine receptor interaction	112	0.66%	Ko 04060
22	Viral carcinogenesis	112	0.66%	Ko 05203
23	Rap1 signaling pathway	110	0.65%	Ko 04015
24	Transcriptional misregulation in cancer	110	0.65%	Ko 05202
25	Regulation of actin cytoskeleton	109	0.64%	Ko 04810
26	Alzheimer disease	108	0.64%	Ko 05010
27	Focal adhesion	105	0.62%	Ko 04510
28	Human immunodeficiency virus 1 infection	105	0.62%	Ko 05170
29	Pathogenic Escherichia coli infection	104	0.61%	Ko 05130
30	Herpes simplex virus 1 infection	104	0.61%	Ko 05168
31	Spliceosome	103	0.61%	Ko 03040
32	Ubiquitin mediated proteolysis	103	0.61%	Ko 04120
33	Non-alcoholic fatty liver disease (NAFLD)	99	0.58%	Ko 04932
34	Kaposi sarcoma-associated herpesvirus infection	99	0.58%	Ko 05167
35	Ribosome	98	0.58%	Ko 03010
36	Autophagy - animal	98	0.58%	Ko 04140
37	Hepatitis B	98	0.58%	Ko 05161
38	mTOR signaling pathway	95	0.56%	Ko 04150

#	Pathway	Number of contigs	Percent of contigs	Pathway ID
39	Tuberculosis	94	0.55%	Ko 05152
40	Lysosome	93	0.55%	Ko 04142
41	NOD-like receptor signaling pathway	93	0.55%	Ko 04621
42	Cellular senescence	89	0.52%	Ko 04218
43	Apoptosis	88	0.52%	Ko 04210
44	Tight junction	87	0.51%	Ko 04530
45	Influenza A	86	0.51%	Ko 05164
46	Hepatocellular carcinoma	86	0.51%	Ko 05225
47	Hepatitis C	85	0.50%	Ko 05160
48	Parkinson disease	84	0.50%	Ko 05012
49	Neuroactive ligand-receptor interaction	83	0.49%	Ko 04080
50	Cell cycle	82	0.48%	Ko 04110
51	Fluid shear stress and atherosclerosis	81	0.48%	Ko 05418
52	Carbon metabolism	80	0.47%	Ko 01200
53	Yersinia infection	80	0.47%	Ko 05135
54	Oxidative phosphorylation	80	0.47%	Ko00190
55	Chemokine signaling pathway	79	0.47%	Ko 04062
56	JAK-STAT signaling pathway	79	0.47%	Ko 04630
57	Measles	79	0.47%	Ko 05162
58	cAMP signaling pathway	78	0.46%	Ko 04024
59	Hippo signaling pathway	77	0.45%	Ko 04390
60	cGMP-PKG signaling pathway	76	0.45%	Ko 04022
61	FoxO signaling pathway	75	0.44%	Ko 04068
62	Necroptosis	75	0.44%	Ko 04217
63	Gastric cancer	75	0.44%	Ko 05226
64	Axon guidance	74	0.44%	Ko 04360
65	Thyroid hormone signaling pathway	74	0.44%	Ko 04919
66	Breast cancer	73	0.43%	Ko 05224
67	Wnt signaling pathway	72	0.42%	Ko 04310
68	Osteoclast differentiation	72	0.42%	Ko 04380
69	Calcium signaling pathway	71	0.42%	Ko 04020
70	Phospholipase D signaling pathway	71	0.42%	Ko 04072
71	TNF signaling pathway	71	0.42%	Ko 04668
72	Insulin signaling pathway	70	0.41%	Ko 04910
73	Cushing syndrome	70	0.41%	Ko 04934
74	NF-kappa B signaling pathway	69	0.41%	Ko 04064
75	Phagosome	69	0.41%	Ko 04145
76	AMPK signaling pathway	69	0.41%	Ko 04152
77	Signaling pathways regulating pluripotency of stem cells	68	0.40%	Ko 04550
78	Purine metabolism	67	0.39%	Ko 00230
79	Platelet activation	66	0.39%	Ko 04611
80	Peroxisome	65	0.38%	Ko 04146

#	Pathway	Number of contigs	Percent of contigs	Pathway ID
81	Ribosome biogenesis in eukaryotes	64	0.38%	Ko 03008
82	Neurotrophin signaling pathway	64	0.38%	Ko 04722
83	Th17 cell differentiation	61	0.36%	Ko 04659
84	Toxoplasmosis	61	0.36%	Ko 05145
85	Sphingolipid signaling pathway	60	0.35%	Ko 04071
86	Apelin signaling pathway	60	0.35%	Ko 04371
87	Cell adhesion molecules (CAMs)	60	0.35%	Ko 04514
88	Leukocyte transendothelial migration	60	0.35%	Ko 04670
89	Oxytocin signaling pathway	60	0.35%	Ko 04921
90	Prostate cancer	60	0.35%	Ko 05215
91	Parathyroid hormone synthesis, secretion and action	59	0.35%	Ko 04928
92	AGE-RAGE signaling pathway in diabetic complications	59	0.35%	Ko 04933
93	HIF-1 signaling pathway	58	0.34%	Ko 04066
94	T cell receptor signaling pathway	58	0.34%	Ko 04660
95	Retrograde endocannabinoid signaling	58	0.34%	Ko 04723
96	Colorectal cancer	58	0.34%	Ko 05210
97	Insulin resistance	57	0.34%	Ko 04931
98	Chagas disease (American trypanosomiasis)	57	0.34%	Ko 05142
99	Complement and coagulation cascades	56	0.33%	Ko 04610
100	Endocrine resistance	55	0.32%	Ko 01522
101	RNA degradation	55	0.32%	Ko 03018
102	C-type lectin receptor signaling pathway	55	0.32%	Ko 04625
103	IL-17 signaling pathway	55	0.32%	Ko 04657
104	Small cell lung cancer	55	0.32%	Ko 05222
105	EGFR tyrosine kinase inhibitor resistance	54	0.32%	Ko 01521
106	mRNA surveillance pathway	54	0.32%	Ko 03015
107	Adherens junction	54	0.32%	Ko 04520
108	Pancreatic cancer	54	0.32%	Ko 05212
109	Vascular smooth muscle contraction	53	0.31%	Ko 04270
110	TGF-beta signaling pathway	53	0.31%	Ko 04350
111	Toll-like receptor signaling pathway	53	0.31%	Ko 04620
112	Growth hormone synthesis, secretion and action	53	0.31%	Ko 04935
113	Longevity regulating pathway	52	0.31%	Ko 04211
114	Chronic myeloid leukemia	52	0.31%	Ko 05220
115	PD-L1 expression and PD-1 checkpoint pathway in cancer	52	0.31%	Ko 05235
116	Glycerophospholipid metabolism	50	0.29%	Ko 00564
117	Glucagon signaling pathway	50	0.29%	Ko 04922
118	Choline metabolism in cancer	50	0.29%	Ko 05231
119	Cell cycle - yeast	49	0.29%	Ko 04111
120	Autophagy - yeast	49	0.29%	Ko 04138
121	Fc gamma R-mediated phagocytosis	49	0.29%	Ko 04666
122	Estrogen signaling pathway	49	0.29%	Ko 04915

#	Pathway	Number of contigs	Percent of contigs	Pathway ID
123	Relaxin signaling pathway	49	0.29%	Ko 04926
124	Bacterial invasion of epithelial cells	49	0.29%	Ko 05100
125	p53 signaling pathway	48	0.28%	Ko 04115
126	Mitophagy - animal	48	0.28%	Ko 04137
127	Adrenergic signaling in cardiomyocytes	48	0.28%	Ko 04261
128	ErbB signaling pathway	47	0.28%	Ko 04012
129	MAPK signaling pathway - fly	47	0.28%	Ko 04013
130	Th1 and Th2 cell differentiation	47	0.28%	Ko 04658
131	Epithelial cell signaling in Helicobacter pylori infection	47	0.28%	Ko 05120
132	Phosphatidylinositol signaling system	46	0.27%	Ko 04070
133	Hematopoietic cell lineage	46	0.27%	Ko 04640
134	Renal cell carcinoma	46	0.27%	Ko 05211
135	Lysine degradation	45	0.27%	Ko 00310
136	Inositol phosphate metabolism	45	0.27%	Ko 00562
137	Amoebiasis	45	0.27%	Ko 05146
138	Biosynthesis of amino acids	44	0.26%	Ko 01230
139	B cell receptor signaling pathway	44	0.26%	Ko 04662
140	PPAR signaling pathway	43	0.25%	Ko 03320
141	Oocyte meiosis	43	0.25%	Ko 04114
142	Acute myeloid leukemia	43	0.25%	Ko 05221
143	Rheumatoid arthritis	43	0.25%	Ko 05323
144	Leishmaniasis	42	0.25%	Ko 05140
145	Axon regeneration	41	0.24%	Ko 04361
146	Natural killer cell mediated cytotoxicity	41	0.24%	Ko 04650
147	Prolactin signaling pathway	41	0.24%	Ko 04917
148	Cholesterol metabolism	41	0.24%	Ko 04979
149	Pertussis	41	0.24%	Ko 05133
150	Valine, leucine and isoleucine degradation	40	0.24%	Ko 00280
151	Proteasome	40	0.24%	Ko 03050
152	RIG-I-like receptor signaling pathway	40	0.24%	Ko 04622
153	GnRH signaling pathway	40	0.24%	Ko 04912
154	Adipocytokine signaling pathway	40	0.24%	Ko 04920
155	Alcoholism	40	0.24%	Ko 05034
156	Salmonella infection	40	0.24%	Ko 05132
157	Non-small cell lung cancer	40	0.24%	Ko 05223
158	Central carbon metabolism in cancer	40	0.24%	Ko 05230
159	Fatty acid metabolism	39	0.23%	Ko 01212
160	Fanconi anemia pathway	39	0.23%	Ko 03460
161	Meiosis - yeast	39	0.23%	Ko 04113
162	Longevity regulating pathway - worm	39	0.23%	Ko 04212
163	Pancreatic secretion	39	0.23%	Ko 04972
164	Endometrial cancer	39	0.23%	Ko 05213

#	Pathway	Number of contigs	Percent of contigs	Pathway ID
165	Hypertrophic cardiomyopathy (HCM)	39	0.23%	Ko 05410
166	Dilated cardiomyopathy (DCM)	39	0.23%	Ko 05414
167	Platinum drug resistance	38	0.22%	Ko 01524
168	Viral protein interaction with cytokine and cytokine receptor	38	0.22%	Ko 04061
169	Apoptosis - fly	38	0.22%	Ko 04214
170	ECM-receptor interaction	38	0.22%	Ko 04512
171	Glioma	38	0.22%	Ko 05214
172	Melanoma	38	0.22%	Ko 05218
173	Nucleotide excision repair	37	0.22%	Ko 03420
174	Dopaminergic synapse	37	0.22%	Ko 04728
175	Inflammatory mediator regulation of TRP channels	37	0.22%	Ko 04750
176	Progesterone-mediated oocyte maturation	37	0.22%	Ko 04914
177	N-Glycan biosynthesis	36	0.21%	Ko 00510
178	Bile secretion	36	0.21%	Ko 04976
179	Legionellosis	36	0.21%	Ko 05134
180	Cysteine and methionine metabolism	35	0.21%	Ko 00270
181	Amino sugar and nucleotide sugar metabolism	35	0.21%	Ko 00520
182	Hippo signaling pathway - fly	35	0.21%	Ko 04391
183	Cytosolic DNA-sensing pathway	35	0.21%	Ko 04623
184	Protein digestion and absorption	35	0.21%	Ko 04974
185	Arrhythmogenic right ventricular cardiomyopathy (ARVC)	35	0.21%	Ko 05412
186	Pyrimidine metabolism	34	0.20%	Ko 00240
187	Antigen processing and presentation	34	0.20%	Ko 04612
188	Aldosterone synthesis and secretion	34	0.20%	Ko 04925
189	Glycine, serine and threonine metabolism	33	0.19%	Ko 00260
190	Melanogenesis	33	0.19%	Ko 04916
191	Glycolysis / Gluconeogenesis	32	0.19%	Ko 00010
192	DNA replication	32	0.19%	Ko 03030
193	Sphingolipid metabolism	31	0.18%	Ko 00600
194	Basal transcription factors	31	0.18%	Ko 03022
195	Longevity regulating pathway - multiple species	31	0.18%	Ko 04213
196	Gap junction	31	0.18%	Ko 04540
197	Fc epsilon RI signaling pathway	31	0.18%	Ko 04664
198	Serotonergic synapse	31	0.18%	Ko 04726
199	Basal cell carcinoma	31	0.18%	Ko 05217
200	Systemic lupus erythematosus	31	0.18%	Ko 05322
201	Synaptic vesicle cycle	30	0.18%	Ko 04721
202	Amyotrophic lateral sclerosis (ALS)	30	0.18%	Ko 05014
203	Inflammatory bowel disease (IBD)	30	0.18%	Ko 05321
204	Arginine and proline metabolism	29	0.17%	Ko 00330
205	Glycerolipid metabolism	29	0.17%	Ko 00561
206	Aminoacyl-tRNA biosynthesis	29	0.17%	Ko 00970

#	Pathway	Number of contigs	Percent of contigs	Pathway ID
207	Hedgehog signaling pathway	29	0.17%	Ko 04340
208	VEGF signaling pathway	29	0.17%	Ko 04370
209	Renin secretion	29	0.17%	Ko 04924
210	Viral myocarditis	29	0.17%	Ko 05416
211	Tryptophan metabolism	28	0.17%	Ko 00380
212	Glutamatergic synapse	28	0.17%	Ko 04724
213	Staphylococcus aureus infection	28	0.17%	Ko 05150
214	Thyroid cancer	28	0.17%	Ko 05216
215	Various types of N-glycan biosynthesis	27	0.16%	Ko 00513
216	Base excision repair	27	0.16%	Ko 03410
217	Homologous recombination	27	0.16%	Ko 03440
218	Ferroptosis	27	0.16%	Ko 04216
219	Notch signaling pathway	27	0.16%	Ko 04330
220	Cholinergic synapse	27	0.16%	Ko 04725
221	Insulin secretion	27	0.16%	Ko 04911
222	Vibrio cholerae infection	27	0.16%	Ko 05110
223	Bladder cancer	27	0.16%	Ko 05219
224	Glutathione metabolism	26	0.15%	Ko 00480
225	Propanoate metabolism	26	0.15%	Ko 00640
226	Malaria	26	0.15%	Ko 05144
227	Fatty acid degradation	25	0.15%	Ko 00071
228	Glyoxylate and dicarboxylate metabolism	25	0.15%	Ko 00630
229	Drug metabolism - other enzymes	25	0.15%	Ko 00983
230	ABC transporters	25	0.15%	Ko 02010
231	RNA polymerase	25	0.15%	Ko 03020
232	SNARE interactions in vesicular transport	25	0.15%	Ko 04130
233	Cardiac muscle contraction	25	0.15%	Ko 04260
234	Thyroid hormone synthesis	25	0.15%	Ko 04918
235	Regulation of lipolysis in adipocytes	25	0.15%	Ko 04923
236	Cortisol synthesis and secretion	25	0.15%	Ko 04927
237	Pyruvate metabolism	24	0.14%	Ko 00620
238	Toll and Imd signaling pathway	24	0.14%	Ko 04624
239	Primary immunodeficiency	24	0.14%	Ko 05340
240	Nicotinate and nicotinamide metabolism	23	0.14%	Ko 00760
241	Antifolate resistance	23	0.14%	Ko 01523
242	Circadian entrainment	23	0.14%	Ko 04713
243	Salivary secretion	23	0.14%	Ko 04970
244	Prion diseases	23	0.14%	Ko 05020
245	Citrate cycle (TCA cycle)	22	0.13%	Ko 00020
246	Fructose and mannose metabolism	22	0.13%	Ko 00051
247	Arachidonic acid metabolism	22	0.13%	Ko 00590
248	Porphyrin and chlorophyll metabolism	22	0.13%	Ko 00860

#	Pathway	Number of contigs	Percent of contigs	Pathway ID
249	Autophagy - other	22	0.13%	Ko 04136
250	Long-term potentiation	22	0.13%	Ko 04720
251	Vasopressin-regulated water reabsorption	22	0.13%	Ko 04962
252	Amphetamine addiction	22	0.13%	Ko 05031
253	Alanine, aspartate and glutamate metabolism	21	0.12%	Ko 00250
254	Glycosylphosphatidylinositol (GPI)-anchor biosynthesis	21	0.12%	Ko 00563
255	Apoptosis - multiple species	21	0.12%	Ko 04215
256	Circadian rhythm	21	0.12%	Ko 04710
257	Long-term depression	21	0.12%	Ko 04730
258	Endocrine and other factor-regulated calcium reabsorption	21	0.12%	Ko 04961
259	Mineral absorption	21	0.12%	Ko 04978
260	Morphine addiction	21	0.12%	Ko 05032
261	Pentose phosphate pathway	20	0.12%	Ko 00030
262	beta-Alanine metabolism	20	0.12%	Ko 00410
263	Ether lipid metabolism	20	0.12%	Ko 00565
264	Gastric acid secretion	20	0.12%	Ko 04971
265	African trypanosomiasis	20	0.12%	Ko 05143
266	Steroid biosynthesis	19	0.11%	Ko 00100
267	Starch and sucrose metabolism	19	0.11%	Ko 00500
268	Retinol metabolism	19	0.11%	Ko 00830
269	Terpenoid backbone biosynthesis	19	0.11%	Ko 00900
270	Mismatch repair	19	0.11%	Ko 03430
271	GABAergic synapse	19	0.11%	Ko 04727
272	Type II diabetes mellitus	19	0.11%	Ko 04930
273	Fat digestion and absorption	19	0.11%	Ko 04975
274	Protein export	18	0.11%	Ko 03060
275	Intestinal immune network for IgA production	18	0.11%	Ko 04672
276	Ovarian steroidogenesis	18	0.11%	Ko 04913
277	Vitamin digestion and absorption	18	0.11%	Ko 04977
278	Cocaine addiction	18	0.11%	Ko 05030
279	Galactose metabolism	17	0.10%	Ko 00052
280	Steroid hormone biosynthesis	17	0.10%	Ko 00140
281	MAPK signaling pathway - yeast	17	0.10%	Ko 04011
282	Mitophagy - yeast	17	0.10%	Ko 04139
283	Hedgehog signaling pathway - fly	17	0.10%	Ko 04341
284	GnRH secretion	17	0.10%	Ko 04929
285	Pentose and glucuronate interconversions	16	0.09%	Ko 00040
286	Fatty acid elongation	16	0.09%	Ko 00062
287	Tyrosine metabolism	16	0.09%	Ko 00350
288	Glycosaminoglycan biosynthesis - heparan sulfate / heparin	16	0.09%	Ko 00534
289	Folate biosynthesis	16	0.09%	Ko 00790
290	Aldosterone-regulated sodium reabsorption	16	0.09%	Ko 04960

#	Pathway	Number of contigs	Percent of contigs	Pathway ID
291	Mannose type O-glycan biosynthesis	15	0.09%	Ko 00515
292	Glycosaminoglycan biosynthesis - chondroitin sulfate / dermatan sulfate	15	0.09%	Ko 00532
293	Methane metabolism	15	0.09%	Ko 00680
294	Carbon fixation in photosynthetic organisms	15	0.09%	Ko 00710
295	Biosynthesis of unsaturated fatty acids	15	0.09%	Ko 01040
296	Hippo signaling pathway - multiple species	15	0.09%	Ko 04392
297	Other glycan degradation	14	0.08%	Ko 00511
298	Other types of O-glycan biosynthesis	14	0.08%	Ko 00514
299	Butanoate metabolism	14	0.08%	Ko 00650
300	One carbon pool by folate	14	0.08%	Ko 00670
301	Dorso-ventral axis formation	14	0.08%	Ko 04320
302	Type I diabetes mellitus	14	0.08%	Ko 04940
303	Chemical carcinogenesis	14	0.08%	Ko 05204
304	Fatty acid biosynthesis	13	0.08%	Ko 00061
305	Glycosaminoglycan degradation	13	0.08%	Ko 00531
306	Maturity onset diabetes of the young	13	0.08%	Ko 04950
307	Collecting duct acid secretion	13	0.08%	Ko 04966
308	Carbohydrate digestion and absorption	13	0.08%	Ko 04973
309	Allograft rejection	13	0.08%	Ko 05330
310	Arginine biosynthesis	12	0.07%	Ko 00220
311	Histidine metabolism	12	0.07%	Ko 00340
312	Non-homologous end-joining	12	0.07%	Ko 03450
313	Graft-versus-host disease	12	0.07%	Ko 05332
314	Primary bile acid biosynthesis	11	0.06%	Ko 00120
315	Pantothenate and CoA biosynthesis	11	0.06%	Ko 00770
316	Metabolism of xenobiotics by cytochrome P450	11	0.06%	Ko 00980
317	2-Oxocarboxylic acid metabolism	11	0.06%	Ko 01210
318	Renin-angiotensin system	11	0.06%	Ko 04614
319	Olfactory transduction	11	0.06%	Ko 04740
320	Autoimmune thyroid disease	11	0.06%	Ko 05320
321	Ubiquinone and other terpenoid-quinone biosynthesis	10	0.06%	Ko 00130
322	Phenylalanine metabolism	10	0.06%	Ko 00360
323	Selenocompound metabolism	10	0.06%	Ko 00450
324	Glycosphingolipid biosynthesis - lacto and neolacto series	10	0.06%	Ko 00601
325	Carbon fixation pathways in prokaryotes	10	0.06%	Ko 00720
326	Proximal tubule bicarbonate reclamation	10	0.06%	Ko 04964
327	Glycosaminoglycan biosynthesis - keratan sulfate	9	0.05%	Ko 00533
328	Glycosphingolipid biosynthesis - ganglio series	9	0.05%	Ko 00604
329	Two-component system	9	0.05%	Ko 02020
330	Phototransduction - fly	9	0.05%	Ko 04745
331	Ascorbate and aldarate metabolism	8	0.05%	Ko 00053

#	Pathway	Number of contigs	Percent of contigs	Pathway ID
332	alpha-Linolenic acid metabolism	8	0.05%	Ko 00592
333	Sulfur metabolism	8	0.05%	Ko 00920
334	Drug metabolism - cytochrome P450	8	0.05%	Ko 00982
335	Sulfur relay system	8	0.05%	Ko 04122
336	Circadian rhythm - fly	8	0.05%	Ko 04711
337	Taurine and hypotaurine metabolism	7	0.04%	Ko 00430
338	Mucin type O-glycan biosynthesis	7	0.04%	Ko 00512
339	Linoleic acid metabolism	7	0.04%	Ko 00591
340	Glycosphingolipid biosynthesis - globo and isoglobo series	7	0.04%	Ko 00603
341	Thiamine metabolism	7	0.04%	Ko 00730
342	Riboflavin metabolism	7	0.04%	Ko 00740
343	Taste transduction	7	0.04%	Ko 04742
344	Asthma	7	0.04%	Ko 05310
345	Streptomycin biosynthesis	6	0.04%	Ko 00521
346	Vitamin B6 metabolism	6	0.04%	Ko 00750
347	Caprolactam degradation	6	0.04%	Ko 00930
348	Isoquinoline alkaloid biosynthesis	6	0.04%	Ko 00950
349	Synthesis and degradation of ketone bodies	5	0.03%	Ko 00072
350	Phosphonate and phosphinate metabolism	5	0.03%	Ko 00440
351	Cyanoamino acid metabolism	5	0.03%	Ko 00460
352	Nitrogen metabolism	5	0.03%	Ko 00910
353	Phenylalanine, tyrosine and tryptophan biosynthesis	4	0.02%	Ko 00400
354	Tropane, piperidine and pyridine alkaloid biosynthesis	4	0.02%	Ko 00960
355	MAPK signaling pathway - plant	4	0.02%	Ko 04016
356	Plant-pathogen interaction	4	0.02%	Ko 04626
357	Caffeine metabolism	3	0.02%	Ko 00232
358	Geraniol degradation	3	0.02%	Ko 00281
359	D-Glutamine and D-glutamate metabolism	3	0.02%	Ko 00471
360	Aminobenzoate degradation	3	0.02%	Ko 00627
361	Styrene degradation	3	0.02%	Ko 00643
362	Biotin metabolism	3	0.02%	Ko 00780
363	Lipoic acid metabolism	3	0.02%	Ko 00785
364	Degradation of aromatic compounds	3	0.02%	Ko 01220
365	Quorum sensing	3	0.02%	Ko 02024
366	Cell cycle - Caulobacter	3	0.02%	Ko 04112
367	Circadian rhythm - plant	3	0.02%	Ko 04712
368	Phototransduction	3	0.02%	Ko 04744
369	Aflatoxin biosynthesis	2	0.01%	Ko 00254
370	Valine, leucine and isoleucine biosynthesis	2	0.01%	Ko 00290
371	Benzoate degradation	2	0.01%	Ko 00362
372	Neomycin, kanamycin and gentamicin biosynthesis	2	0.01%	Ko 00524
373	Chloroalkane and chloroalkene degradation	2	0.01%	Ko 00625

#	Pathway	Number of contigs	Percent of contigs	Pathway ID
374	Sesquiterpenoid and triterpenoid biosynthesis	2	0.01%	Ko 00909
375	Phenylpropanoid biosynthesis	2	0.01%	Ko 00940
376	Betalain biosynthesis	2	0.01%	Ko 00965
377	Bacterial secretion system	2	0.01%	Ko 03070
378	Nicotine addiction	2	0.01%	Ko 05033
379	Cutin, suberine and wax biosynthesis	1	0.01%	Ko 00073
380	Monobactam biosynthesis	1	0.01%	Ko 00261
381	Lysine biosynthesis	1	0.01%	Ko 00300
382	Penicillin and cephalosporin biosynthesis	1	0.01%	Ko 00311
383	Prodigiosin biosynthesis	1	0.01%	Ko 00333
384	Chlorocyclohexane and chlorobenzene degradation	1	0.01%	Ko 00361
385	Fluorobenzoate degradation	1	0.01%	Ko 00364
386	Novobiocin biosynthesis	1	0.01%	Ko 00401
387	D-Arginine and D-ornithine metabolism	1	0.01%	Ko 00472
388	Polyketide sugar unit biosynthesis	1	0.01%	Ko 00523
389	Acarbose and validamycin biosynthesis	1	0.01%	Ko 00525
390	Toluene degradation	1	0.01%	Ko 00623
391	Naphthalene degradation	1	0.01%	Ko 00626
392	Nitrotoluene degradation	1	0.01%	Ko 00633
393	C5-Branched dibasic acid metabolism	1	0.01%	Ko 00660
394	Indole alkaloid biosynthesis	1	0.01%	Ko 00901
395	Limonene and pinene degradation	1	0.01%	Ko 00903
396	Zeatin biosynthesis	1	0.01%	Ko 00908
397	Flavone and flavonol biosynthesis	1	0.01%	Ko 00944
398	Insect hormone biosynthesis	1	0.01%	Ko 00981
399	Biosynthesis of ansamycins	1	0.01%	Ko 01051
400	Biosynthesis of vancomycin group antibiotics	1	0.01%	Ko 01055
401	Biofilm formation - Escherichia coli	1	0.01%	Ko 02026

Supplementary Table S4: Pathway distribution of major pathway categories

Pathway Category	
**Cellular Processes**	
Cell growth and death	602
Cell motility	109
Cellular community - eukaryotes	345
Cellular community - prokaryotes	4
Transport and catabolism	605
**Environmental Information Processing**	
Membrane transport	27
Signal transduction	2007
Signaling molecules and interaction	331
**Genetic Information Processing**	
Folding, sorting and degradation	368
Replication and repair	193
Transcription	159
Translation	368
**Human Diseases**	
Cancers: Overview	902
Cancers: Specific types	843
Cardiovascular diseases	223
Drug resistance: Antineoplastic	170
Endocrine and metabolic diseases	331
Immune diseases	171
Infectious diseases: Bacterial	683
Infectious diseases: Parasitic	251
Infectious diseases: Viral	1202
Neurodegenerative diseases	359
Substance dependence	103
**Metabolism**	
Amino acid metabolism	288
Biosynthesis of other secondary metabolites	34
Carbohydrate metabolism	326
Energy metabolism	133
Global and overview maps	1572
Glycan biosynthesis and metabolism	213
Lipid metabolism	289
Metabolism of cofactors and vitamins	141
Metabolism of other amino acids	77
Metabolism of terpenoids and polyketides	30
Nucleotide metabolism	101
Xenobiotics biodegradation and metabolism	65
**Organismal Systems**	
Aging	122
Circulatory system	126
Development and regeneration	201
Digestive system	265
Endocrine system	909
Environmental adaptation	188
Excretory system	82
Immune system	1045
Nervous system	337
Sensory system	67

Supplementary Figure S5. Full-length gels from PCR results displayed in Figure 4. Red rectangles represent cropping lines. All gels have been run under the same experimental conditions. **1**. GAPDH, **2**. RPL13A, **3**. Ef1a, **4**. SOD1, **5**. CAT, **6**. Hsp70, **7**. HIF-1a, **8**. CYP1A1, **9**. CYP3A, **10**. RXRA X2, **11**. AHR, **12**. Bcl-X, **13**. Bax, **14**. CASP3, **15**. Gadd45A, **16**. TNFAIP3, **17**. IL6, **18**. HLA-DRA, **19**. PPARA, **20**. PPARG.

1.

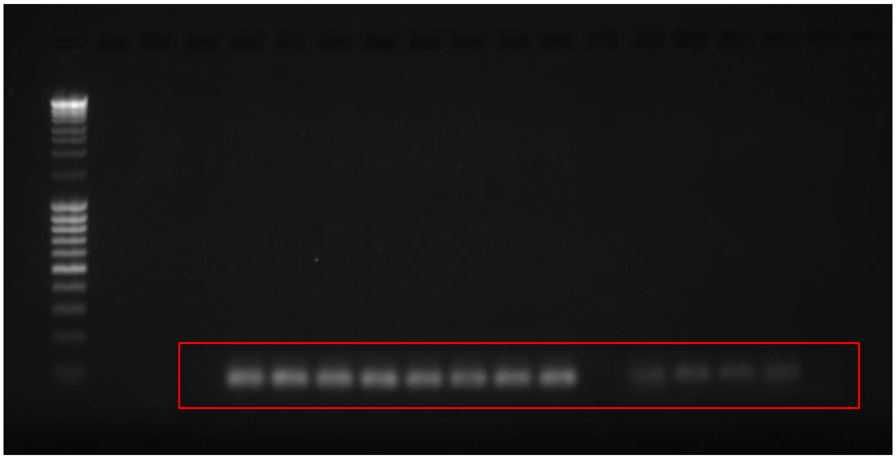

2.

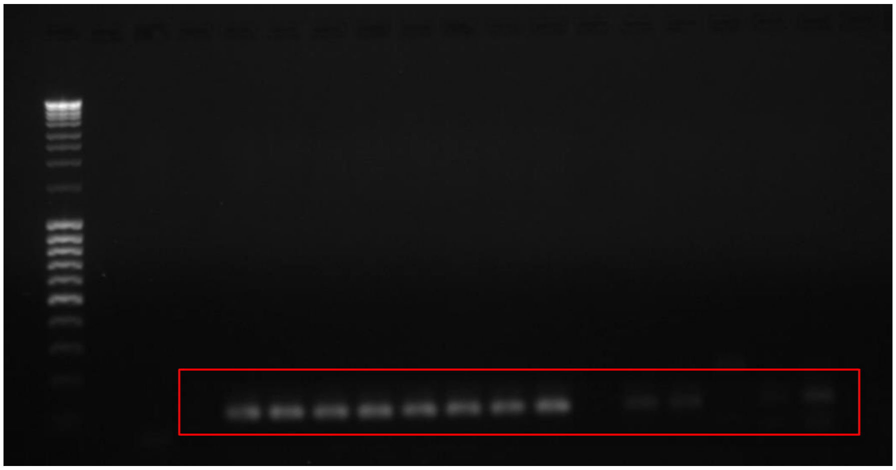

3.

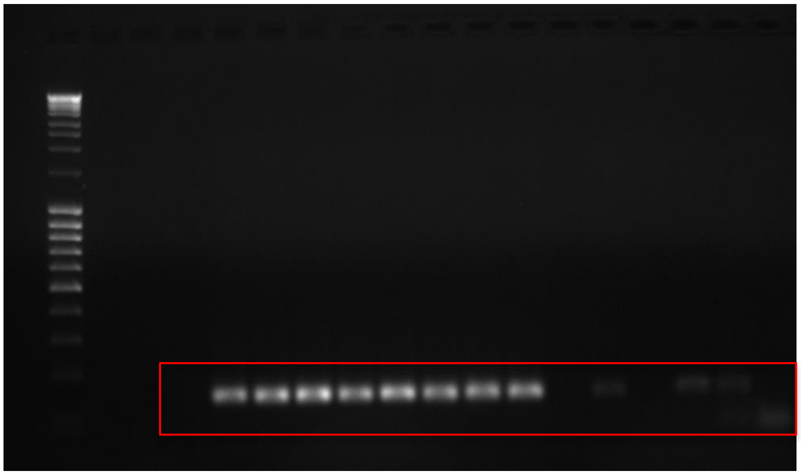

4.

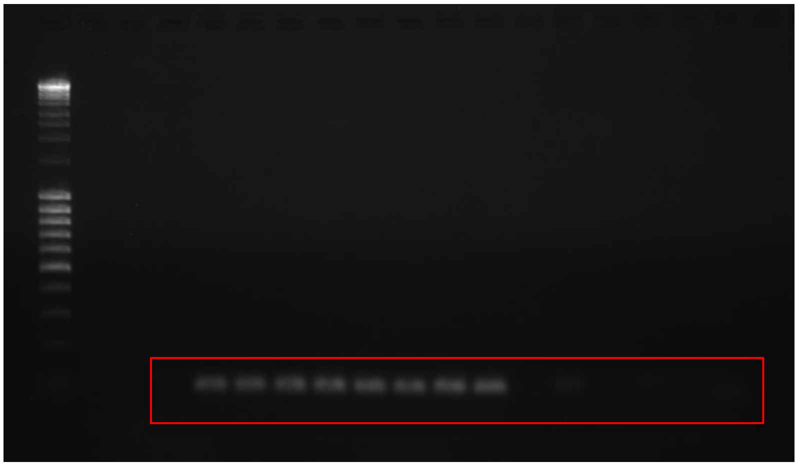

5.

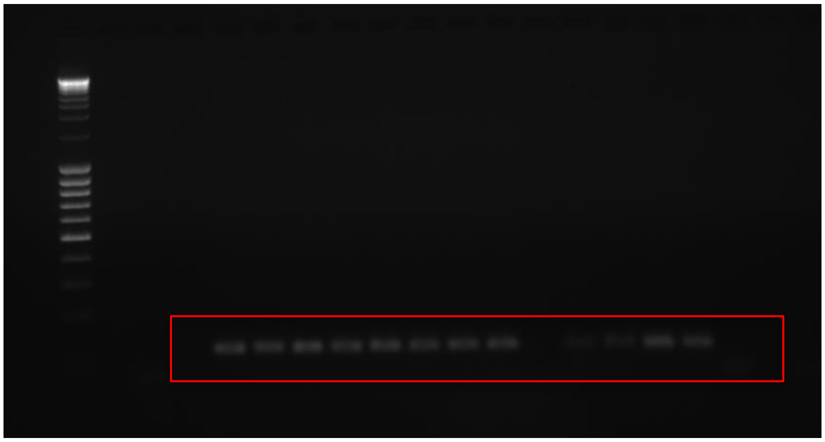

6.

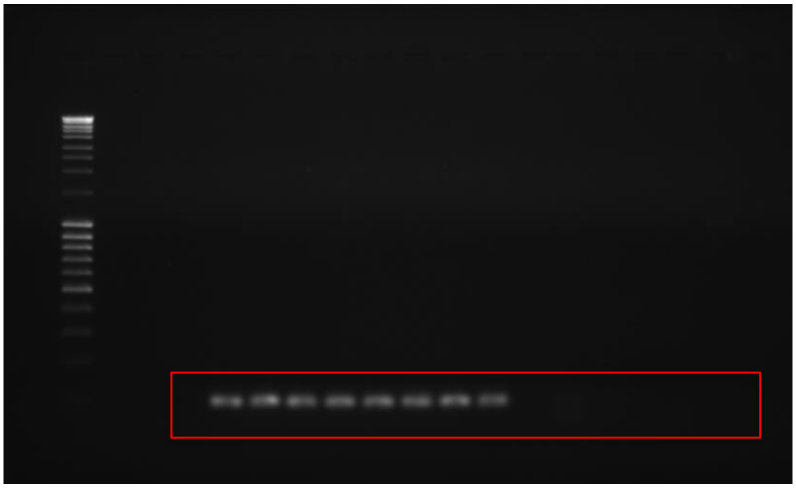

7.

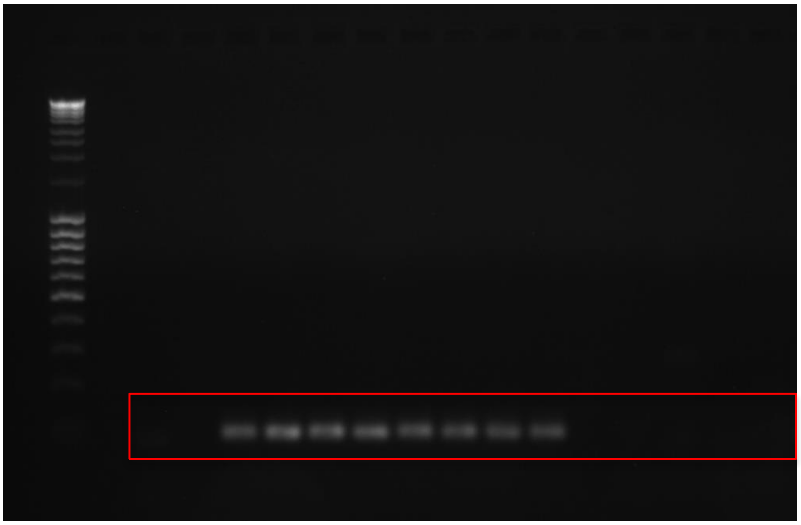

8.

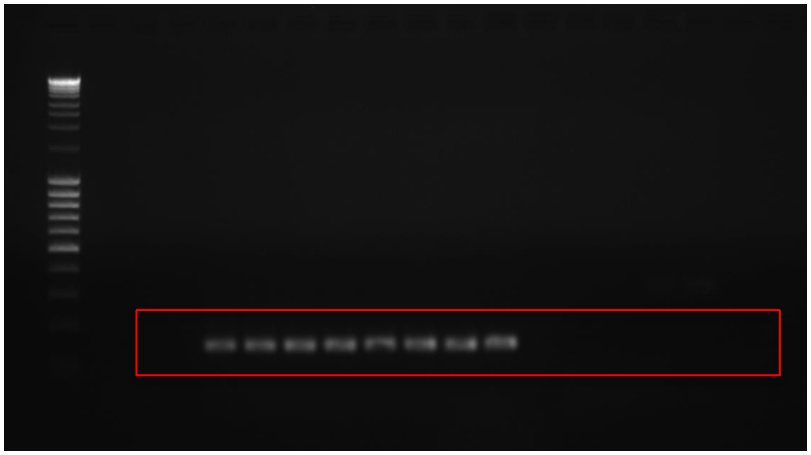

9.

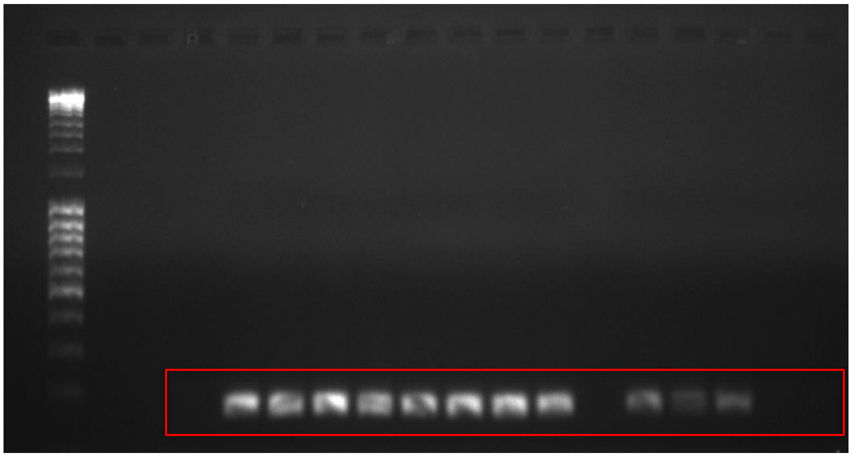

10.

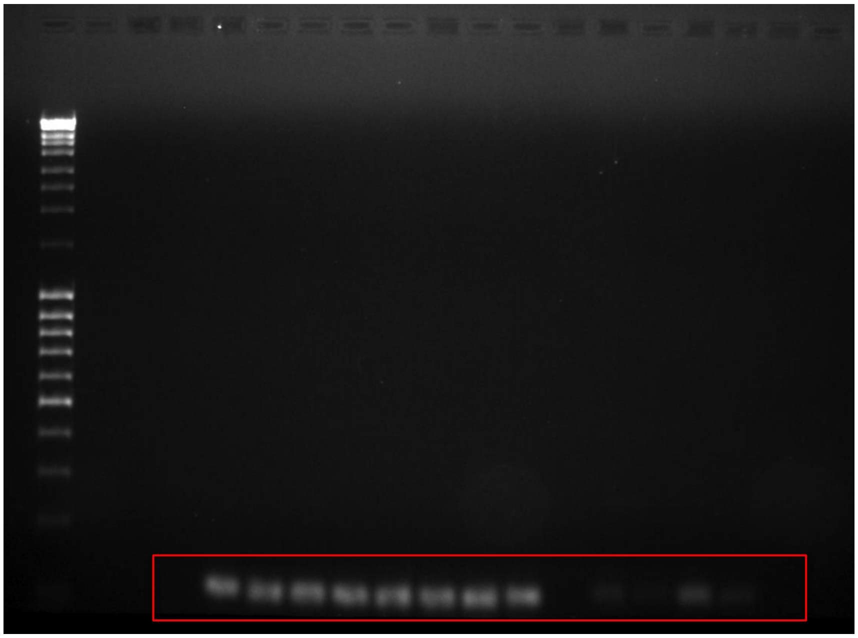

11.

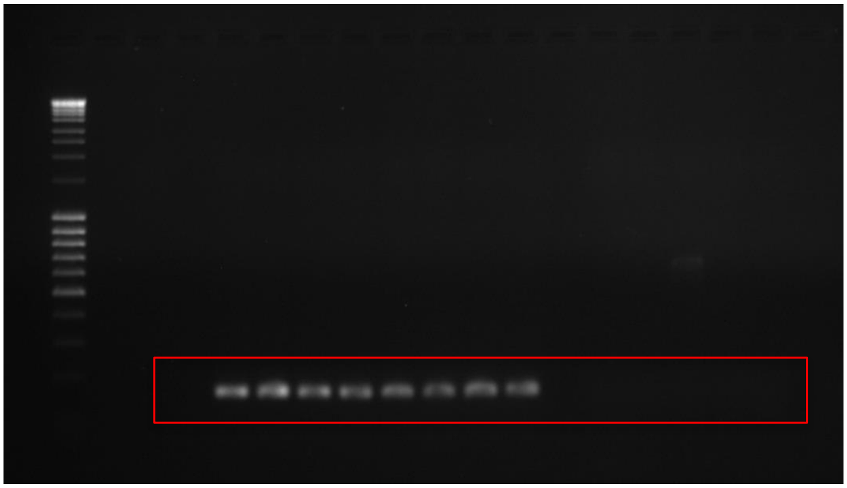

12.

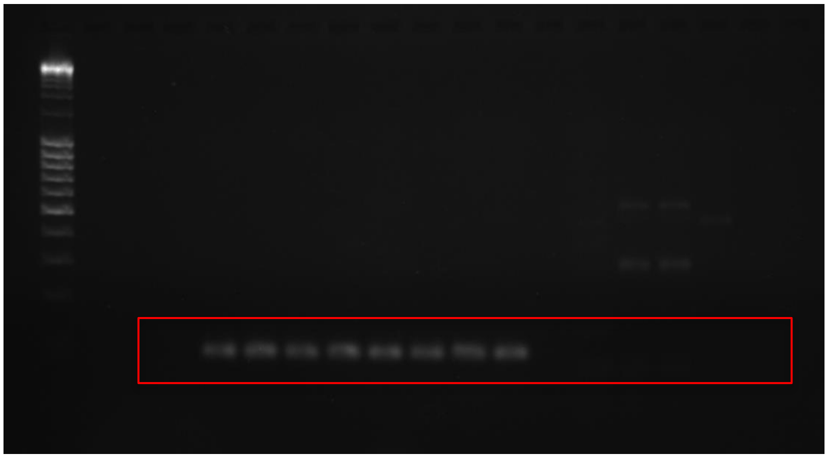

13.

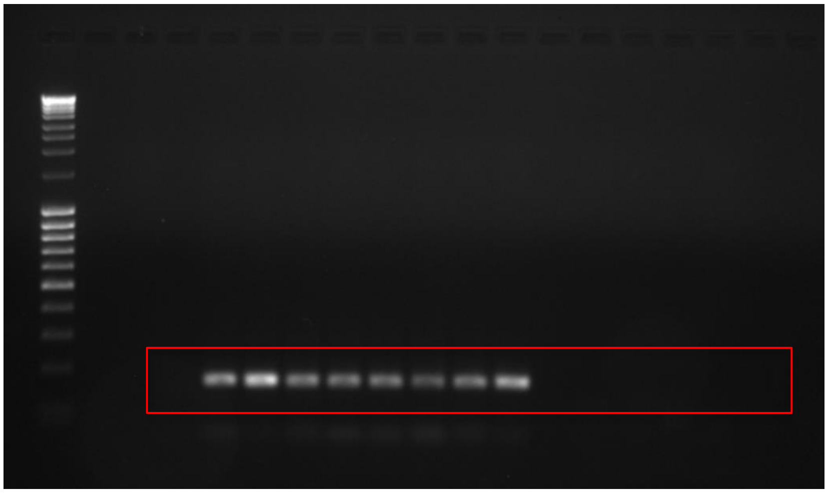

14.

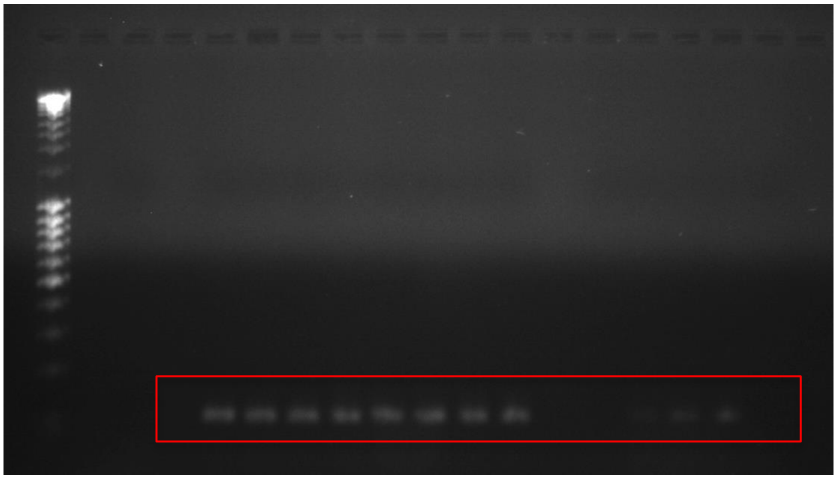

15.

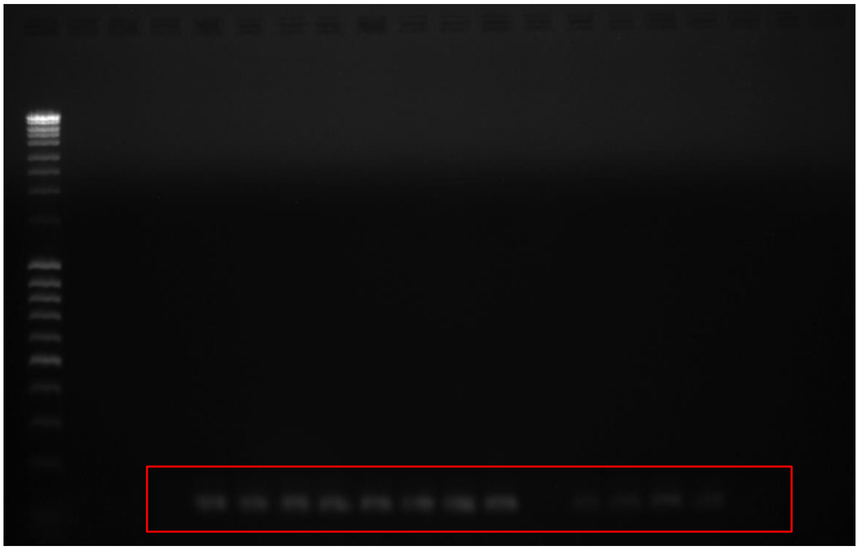

16.

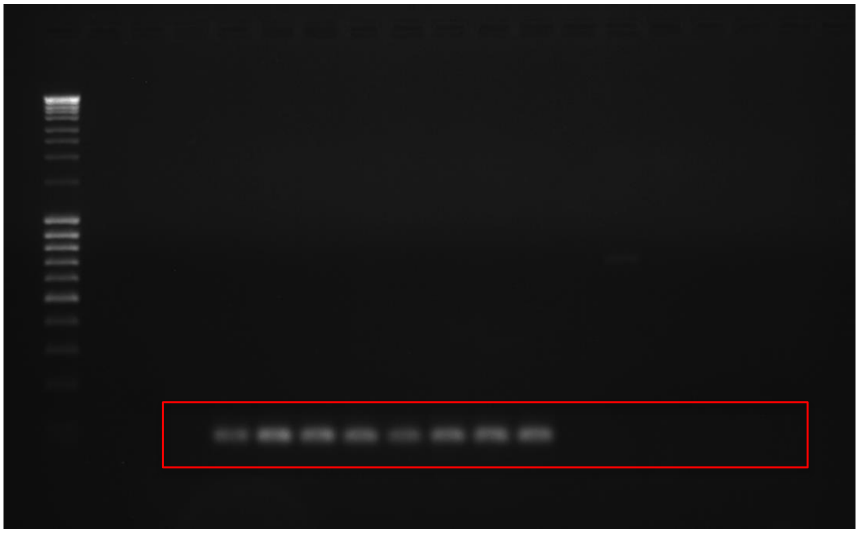

17.

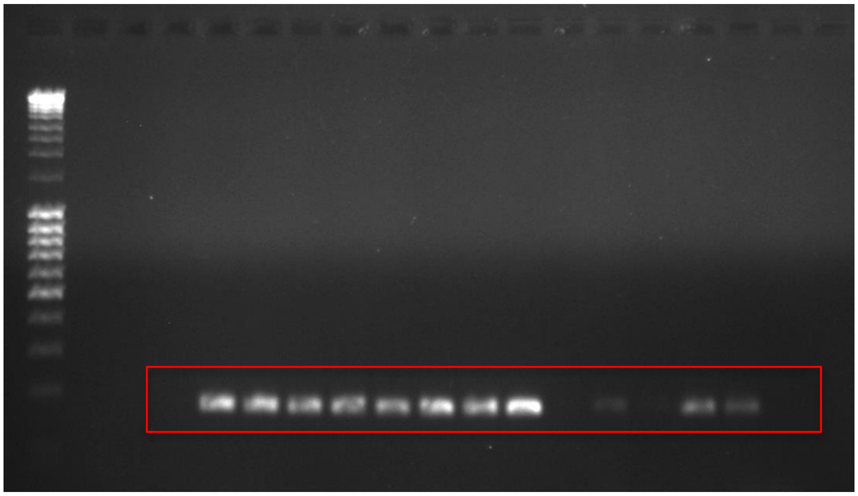

18.

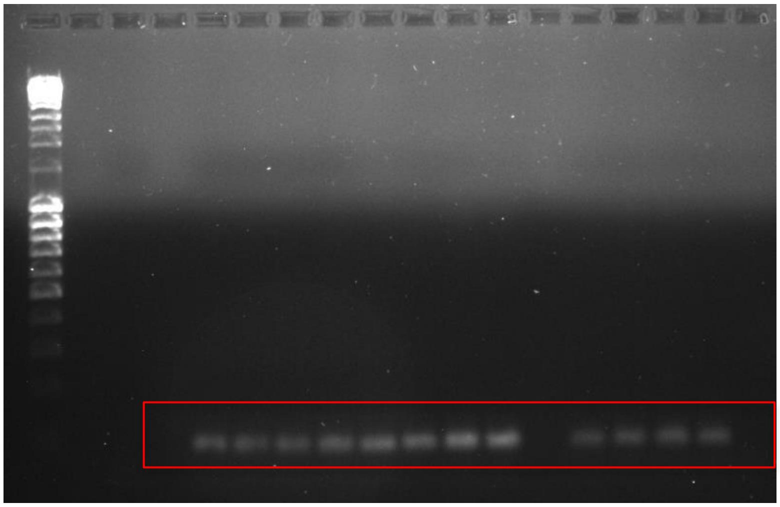

19.

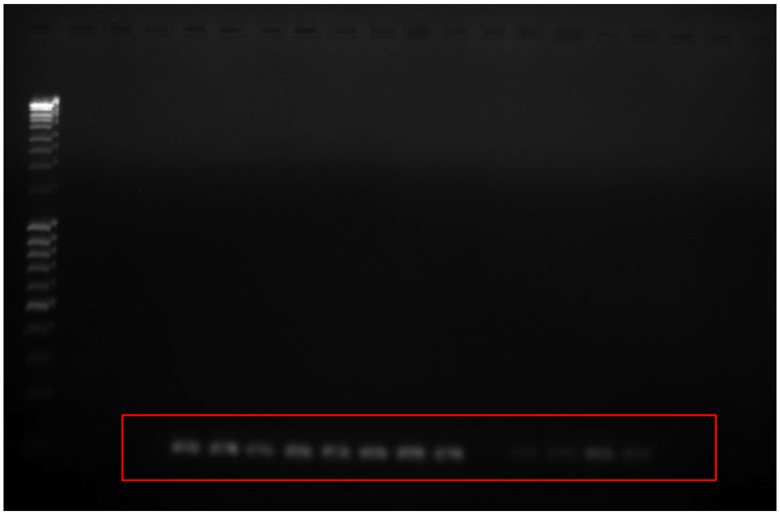

20.

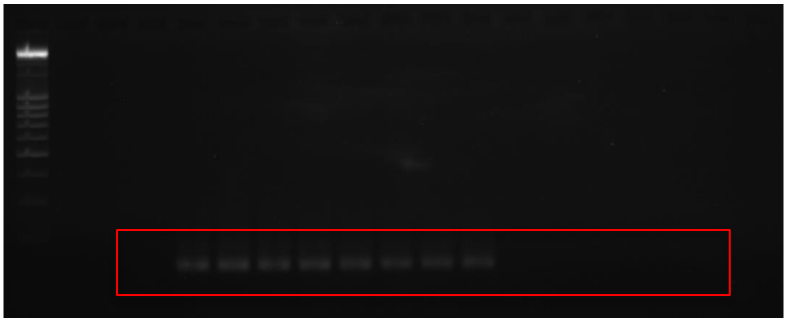

Supplement: Supplementary file 1 — Supplementary information. [file 41598_2020_70902_MOESM1_ESM.pdf]
